# Ecofriendly Filtration of Silver Nanoparticles for Ultrasensitive Surface-Enhanced (Resonance) Raman Spectroscopy-Based Detection

**DOI:** 10.1021/acs.jpcc.4c03837

**Published:** 2024-09-23

**Authors:** Kevin M. Dorney, Nicholas S. Shropshire, Daniel G. Adams, Ashkan Zandi, Joshua Baker, Seth Brittle, Sushil Kanel, Nasrin Hooshmand, Ioana E. Pavel

**Affiliations:** † Department of Chemistry, Wright State University, 3640 Colonel Glenn Hwy., Dayton, Ohio 45435, United States; ‡ Department of Physical and Environmental Sciences, Texas A&M University–Corpus Christi, Corpus Christi, Texas 78412, United States; § School of Electrical and Computer Engineering, Georgia Institute of Technology, North Avenue, Atlanta, Georgia 30332, United States; ∥ UES, 4401 Dayton Xenia Rd, Beavercreek, Ohio 45432, United States

## Abstract

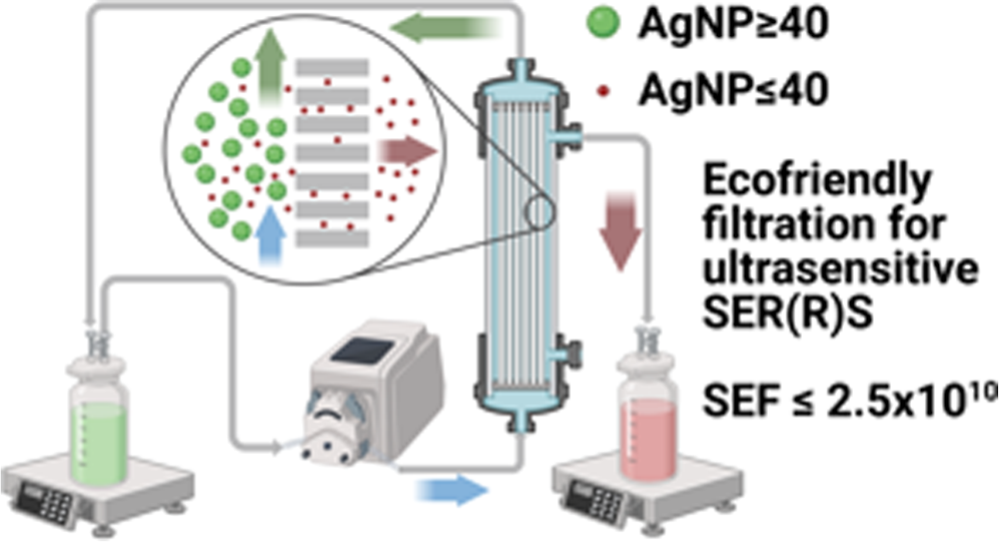

In this study, a widely used colloid of Creighton AgNPs (ORI, 1–100 nm, mostly ≤ 40 nm, ∼10 μg mL^–1^) was rapidly manipulated via tangential flow filtration (TFF) for highly reproducible surface-enhanced (resonance) Raman spectroscopy (SE(R)RS) experiments down to the single-molecule (SM) level. The quasi-spherical AgNPs were size-selected, purified, and concentrated in two TFF fractions of a cutoff diameter of ∼40 nm: AgNP ≤ 40 (∼900 μg mL^–1^) and AgNP ≥ 40 (∼100 μg mL^–1^). The SE(R)S-based sensing capabilities of the two TFF fractions were then tested under pre-resonance (632.8 nm) and resonance (532.1 nm) excitation conditions for rhodamine 6G (R6G, 10^–6^–10^–15^ M). Both TFF isolates, AgNP ≤ 40 and AgNP ≥ 40, were more effective in adsorbing the R6G analyte (≥91%) than the original colloid (≥78%) at submonolayer coverages. Furthermore, the surface enhancement factors (SEF) of the two TFF fractions were markedly superior to those of ORI under all excitation conditions. SERS at 632.8 nm: only AgNP ≥ 40 enabled the detection of R6G at 10^–9^ M and produced the largest SEF (2.1 × 10^6^). SE(R)RS and SM-SERRS at 532.1 nm: AgNP ≥ 40 gave rise to the largest SEF values (2.5 × 10^10^) corresponding to the SM regime down to 10^–15^ M of R6G. Nevertheless, AgNP ≤ 40 compensated for the size-dependence of the electromagnetic enhancements by an increase in the silver concentration, which led to SEF values comparable to those of AgNP ≥ 40 through additional resonance enhancements. TFF resulted into a ∼100-fold increase (AgNP ≤ 40) in the number of negatively charged AgNPs that were available to electrostatically bridge R6G cations and form SERRS “hot-spots” (AgNP-R6G-AgNP) within the focal volume. Evidently, the interplay between AgNP size, AgNP concentration, and excitation wavelength governs the SE(R)RS enhancements. This study demonstrated that TFF can facilitate the ecofriendly isolation of spherical AgNPs of controlled morphological and plasmonic properties for further enhancing their sensing capabilities as SE(R)RS substrates.

## 1 Introduction

Surface-enhanced Raman spectroscopy (SERS) is a powerful sensing technique that has the molecular fingerprinting and multiplex detection capabilities of Raman spectroscopy but significantly lower detection limits.^1−8^ Thus, SERS can overcome the intrinsic low efficiency of the ordinary Raman scattering processes () under aqueous, nondestructive conditions. Surface-enhanced resonance Raman spectroscopy (SERRS) spectra have been reported for “single-molecules” such as small chromophores (e.g., rhodamine 6G-R6G and hemoglobin-Hb) located at the nanosized interstitial sites between metallic nanoparticles (NPs) and outside sharp surface protrusions of metallic nanostructures (known as “hot spots”).^9,10^ Extensive theoretical and experimental research has confirmed that the large signal enhancements observed in SE(R)RS studies are the result of three primary mechanisms: (i) an electromagnetic enhancement (EM) mechanism due to laser-induced excitation of dipoles and multipoles in the metallic nanosurfaces, (ii) a charge transfer mechanism between the vibrational LUMO of the adsorbed molecule and the Fermi electron level of the metal, and (iii) a resonance enhancement (RE) mechanism when the excitation source is coupled to a molecular resonance in the adsorbate.^1−3,11−20^ The EM and RE mechanisms are in general the largest contributors to the SE(R)RS effect.^16,17^ However, metallic SERS nanosubstrates such as silver NPs (AgNPs) can be coupled to thermoelectric semiconductors of gallium nitride to modulate the Fermi level (i.e., dual EM and CM regulation of ∼10-fold larger signals) and expand the SERS-based detection range (e.g., molecules of small Raman scattering cross-sections).^19,20^ Therefore, the choice of a particular nanostructure morphology and excitation wavelength is a key parameter governing the enormous signal enhancements observed in SE(R)RS.

The largest EM enhancements were observed for Ag when compared to other noble metals such as gold and copper.^21,22^ Hence, Ag is routinely utilized as a SE(R)RS substrate in various nanostructured forms, such as colloidal AgNPs.^1−3,23^ The EM enhancement mechanism observed for AgNPs arises from the localized surface plasmon resonance (LSPR) excitations via the interaction with optical laser frequencies, which results in an exponential increase in the magnitude of both the incident and scattered EM field near the AgNP surface.^13,16^ The LSPR observed in metallic NPs has a direct dependence on their size,^24^ shape,^25^ dielectric surroundings,^26^ and aggregation state.^27^ Consequently, specific control of AgNP morphology, dimensionality, and local environment have been utilized to further enhance their SE(R)RS-based sensing capabilities.^28,29^ Recent advancements in synthetic nanotechnology have been employed to achieve precise size and morphological control during AgNP formation using elaborate physicochemical techniques such as chemical vapor deposition,^30^ nanosphere lithography,^8^ and solution-phase seeded-growth mechanisms.^29^ Alternatively, the nanoscale dimensions and size-distribution of AgNPs can be modified postsynthetically using exclusion procedures like size-exclusion chromatography,^31,32^ ultracentrifugation,^32^ fractional crystallization,^33^ and supercritical fluids.^34^ Despite the myriad of techniques available to the synthetic chemist or spectroscopist, nearly all these approaches possess potential shortcomings such as considerable cost and time, low yield, high toxicity of reagents, or uncontrolled aggregation of AgNPs.^8,30−34^ Furthermore, most manipulation techniques for AgNPs require elaborate experimental designs, which may not be easily scaled-up or employed in industrial settings.^8,30−34^

**Figure 1:**
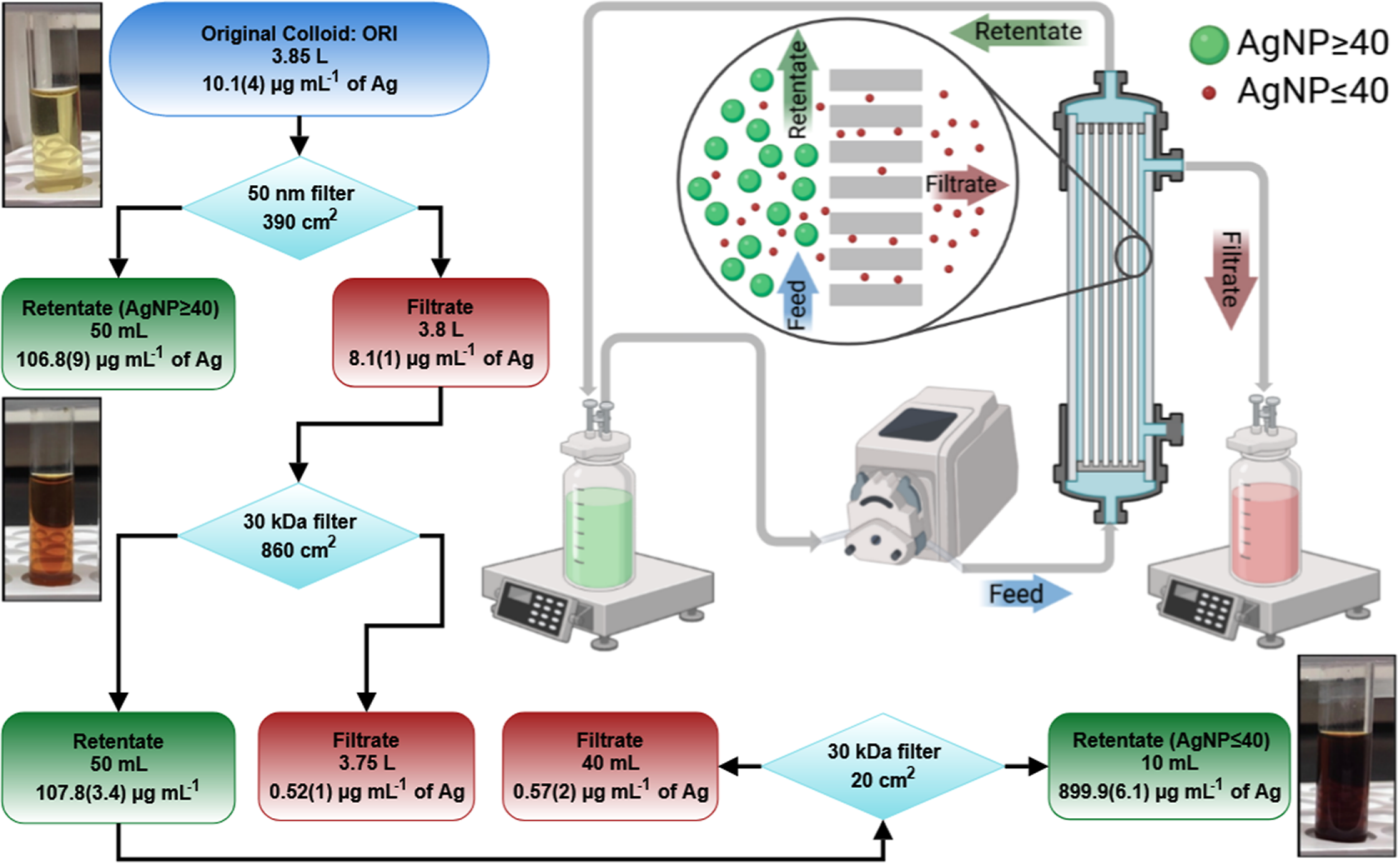
Workflow diagram of the three-step TFF process of a batch of colloidal AgNPs (left) and a simplified diagram of the TFF working principle through a hollow fiber membrane (right). Some objects might be out of scale for illustrative purposes. Visual inspections are shown for the original colloid (ORI) and the two TFF fractions (named AgNP ≤ 40 and AgNP ≥ 40) utilized in the Raman and SE(R)RS experiments. Volumes and concentrations of the TFF fractions are given for each filtration step.

Tangential flow filtration (TFF) or cross-flow filtration stands out as an effective alternative to other size-selection and exclusion techniques for spherical AgNPs because it is ecofriendly as well as time- and cost-effective.^35−38^ In TFF, the size-based separation of particles is driven by the tangential flow across hollow fiber porous membranes (1–1000 kDa) and the resulting pressure difference across the membrane.^35−40^ The semipermeable membrane of a specific pore size separates molecules or particles from a liquid mixture, which is aspirated into the system with the help of a peristaltic pump. The pressure difference between the two sides of the membrane presses a portion of the fluid containing the solid particles against the filter membrane, while the retained sample portion can be recirculated through the TFF system (Figure 1). In contrast to batchwise, dead-end, or direct flow filtration (DFF), TFF can be performed in a continuous flow mode that recirculates the feed stream in a loop until the desired level of separation, concentration, and purification of particles is achieved. TFF is widely used by biopharmaceutical industries due to its high “green” efficiency (nonthermal and 70% less water usage) and over 45% increase in separation productivity (reduced “filter caking” and automated processing).^39,40^ TFF minimizes the “filter cake” characteristic of DFF through the tangential flow to the membrane, which sweeps away individual or aggregated particles that are larger than the pore size and can clog the porous membrane through buildup. These particles are collected on the feed side (the retentate) at the completion of the filtration process. Conversely, particles smaller than the membrane pores are pushed through and gradually collected at each circulation loop (the filtrate). TFF operates at low transmembrane pressures, which ensures gentle particle separation and minimal shear stress. The shear rate can be controlled through the feed flow rate. As a result, the low, constant flow rate prolongs the lifespan of the membrane and prevents the aggregation or damage of sensitive samples.^39^ In our previous work, we demonstrated that the recirculation of a colloidal sample of AgNPs (2 mL–15 L) through the TFF system (Figure S1) can result in the rapid size-selection, purification, and concentration of AgNPs.^35−37^ Furthermore, it did not require the use of additional potentially hazardous functional agents or solvents for controlling the morphology and optical properties of nanoplasmonics. Thus, TFF can significantly reduce the inherent polydispersity of simple colloidal models of AgNPs and thereby transform them into uniform SERS substrates of significantly enhanced reproducibility and sensitivity.^35−37^ In this study, TFF will be applied with a cutoff diameter of ∼40 nm to a widely used colloidal model of Creighton AgNPs (quasi-spherical and unfunctionalized)^35^ that could lead to reproducible SE(R)RS detection events down to the single-molecule (SM) level.

One of the most synthetically simple and well-studied SE(R)RS substrates leading to enormous enhancements with the capability of SM detection events are spherical colloidal AgNPs.^1−3^ Thus, the effects of excitation wavelength, size, and concentration of spherical AgNPs on the SE(R)RS signal enhancement have been an area of intensive study.^29,41−44^ For instance, McFarland et al.^42^ have designed an ingenious scanning wavelength system to show that the SERS enhancement factor (EF) is the greatest when the excitation wavelength is shifted close to the LSPR maximum of adsorbate (benzenethiol)-covered AgNP arrays fabricated by nanosphere lithography. Shao et al.^18^ have also reported largely enhanced SERS signals when tuning the energy of the excitation laser to the energy gap between the Fermi level of the SERS substrate (e.g., graphene oxide coupled to a ferroelectric material) and the LUMO of adsorbate molecules (e.g., R6G). Stamplecoskie et al.^29^ established that spherical AgNPs of ∼50 nm obtained through a seeded growth mechanism yield the largest off-resonance (785 nm) SERS EFs for rhodamine 6G (R6G) molecules. In our previous work,^35^ TFF-fractionated Creighton AgNPs (11 nm in average diameter, narrow size distribution, low aggregation, and 198.7 μg mL^–1^) exhibited a ∼1000-fold increase in surface EFs (SEF) when compared to the original colloid under pre-resonance excitation conditions (632.8 nm) for R6G. However, no further SE(R)RS explorations were carried out to examine the SEF of the TFF-fractionated AgNPs at ultralow analyte concentrations and as a function of their morphology or excitation wavelength.^35−37^

Herein, it is hypothesized that the largest, reproducible SE(R)RS enhancements will be observed for: (1) all TFF fractions when compared to the original Creighton colloid (denoted ORI) and under all laser excitation conditions for R6G (pre-resonance at 632.8 nm and resonance at 532.1 nm), (2) the TFF fractions containing mostly large AgNPs (denoted AgNP ≥ 40) under all excitation conditions and at comparable Ag concentrations, and (3) the TFF fractions mostly consisting of small AgNPs ≤ 40 nm (denoted AgNP ≤ 40) under resonance conditions and enhanced Ag concentrations. To test these hypotheses, the SE(R)RS EFs^36^ will be estimated from the Raman, SE(R)RS, and fluorescence emission spectra of R6G before and after the adsorption the two TFF fractions and ORI of AgNPs. In addition, key physicochemical properties governing the SE(R)RS enhancements of AgNPs (e.g., size, shape, metal concentration, purity/environment, and LSPR) will be characterized by well-established optical spectroscopy and electron imaging techniques. Theoretical simulations using the finite-difference time-domain (FDTD) method will also be performed to guide the selection of the excitation wavelength for optimum SE(R)RS enhancements and to gain a better understanding of the experimental data. FDTD is a well-known technique for its precision in modeling the optical properties of plasmonic NPs of arbitrary sizes and shapes as well as the plasmonic field coupling of NPs.^1^

## 2 Experimental Methods

### 2.1 Chemicals and Materials

Silver nitrate (99% AgNO_3_), sodium borohydride (99% NaBH_4_), rhodamine 6G chloride (99% R6G), potassium bromide (98% KBr), trace metal grade hydrochloric acid (HCl, 37%), optima grade nitric acid (HNO_3_, 67–70%), trace metal grade Ag (1.00 × 10^3^ μg mL^–1^), and trace metal grade sodium (Na, 1.00 × 10^4^ μg mL^–1^) were purchased from Ultrascientific (Na and Ag) and Fisher Scientific (all other reagents). All chemicals were used as received, without further purification. High-purity water (>18 MΩ cm, HQ H_2_O) was utilized throughout the experiments. The hollow-fiber filter modules were purchased from Repligen.

### 2.2 Synthesis of Colloidal Silver Nanoparticles (AgNPs)

Approximately 3.85 L of colloidal AgNPs were synthesized using a modified Creighton method.^35,45^ Briefly, 2.0 mM of NaBH_4_ solution was prepared in 300 mL of ice-cold water (2 ± 1 °C), and 50 mL of 1.0 mM of AgNO_3_ solution (10 ± 2 °C) was slowly added to it with continuous stirring. The reaction was allowed to proceed for 50 min, with moderate stirring, in an ice-bath, and the resulting colloid of AgNPs was stored in a refrigerator at 4 ± 1 °C. The Creighton procedure was carried out in a batch-wise process until the desired volume of colloidal AgNPs was achieved.

### 2.3 Tangential flow filtration of AgNPs

A three-step TFF process (Figures 1 and S1) was employed following our previously developed procedure using a KrosFlo II system (Repligen).^35−37^ In the first step, colloidal AgNPs (denoted ORI) were fractionated using a 50 nm polysulfone (PS, surface area (SA) of 390 cm^2^, ∼ 50 nm) hollow fiber module, yielding a concentrated retentate of AgNPs (denoted AgNP ≥ 40) and a dilute filtrate of AgNPs. In the second and third steps, the resulting 50 nm-filtrate was further fractionated and purified using two 30 kDa modified poly(ether sulfone) hollow fiber membranes (mPES, SA of 860 cm^2^ and then SA of 20 cm^2^, ∼5–6 nm pore sizes). These two steps resulted in a highly concentrated retentate (denoted AgNP ≤ 40) and two 30 kDa diluted filtrates of AgNPs. The TFF process was performed on three independent batches of Creighton AgNPs. Filter membranes were cleaned before and after each filtration step by using a 10% v/v solution of HCl/HNO_3_ for 10 min. Aliquots of the TFF colloidal samples were collected and characterized as outlined below.

### 2.4 Inductively Coupled Plasma-Optical Emission Spectroscopy of AgNPs

Silver (Ag^+^ of 25, 50, 75, 100, 125, and 150 μg L^–1^) standards, sodium (Na^+^ of 100, 200, 400, 500, 600, 800, and 1000 μg L^–1^) standards, and blanks (0 μg L^–1^ of metal) were prepared through serial dilutions from their trace metal grade stock solutions in a 2% HNO_3_ aqueous matrix. Colloidal samples of AgNPs were prepared in triplicate through “cold” and “hot” chemical digestions in 70% HNO_3_, followed by dilutions and storage in a 2% aqueous matrix of HNO_3_.

The total Ag and Na contents of the TFF fractions of colloidal AgNPs were determined by ICP-OES using a sequential Varian 710-ES system (Agilent Technologies) in an axial viewing position. The system was fed compressed liquid argon, and each sample was measured in triplicate. The acquisition parameters were two wavelengths for Ag (328.068 and 338.289 nm), two wavelengths for Na (589.592 and 588.995 nm), a radio frequency power of 1.20 kW, a plasma flow of 15.0 L min^–1^, an auxiliary flow of 1.50 L min^–1^, a nebulizer pressure of 200 kPa, a replicate acquisition time of 15 s, a stabilization time of 40 s, and an uptake delay of 45 s. Once the measurements were completed, the total metal content of each sample (Table S1) was estimated through interpolation from the external calibration curves (Figure S2).

### 2.5 UV–Vis Absorption Spectrophotometry of AgNPs

Aliquots of ORI, AgNP ≥ 40, and AgNP ≤ 40 were diluted with water and placed in a quartz cuvette of a 1 cm path length. The extinction spectra of the colloidal samples were collected using a Cary 50 Bio UV–visible spectrophotometer (Varian, Inc.) at a scan rate of 1200 nm min^–1^. The blank spectra were automatically subtracted, and the spectral resolution was ∼1 nm.

### 2.6 Transmission Electron Microscopy of AgNPs

Aliquots (∼10 μL) of ORI, AgNP ≥ 40, and AgNP ≤ 40 were deposited onto carbon coated copper grids and imaged using a CM200 transmission electron microscopy (TEM) (Phillips) at an accelerating potential of 200 kV. A TEM micrograph analysis was performed in the ImageJ 1.46R software, and average diameters were derived from the area values of over n = 500 AgNPs (Table S2). A single AgNP was defined as a complete and enclosed parameter (circular SA, *A* = π*r*^2^), and an area threshold value of 1.0 nm^2^ was imposed based on the spatial resolution of the TEM micrographs.

### 2.7 Raman and SERS of AgNPs

Raman samples required no preparation: control, blank AgNPs (no R6G) and 10^–5^–10^–14^ M aqueous stock solutions of R6G (no AgNPs) were directly added to quartz cuvettes for Raman analysis. SE(R)RS samples were obtained by adding aliquots of R6G stock solutions to the colloidal samples of AgNPs (ORI, AgNP ≥ 40, and AgNP ≤ 40) to yield final R6G concentrations of 1 × 10^–6^–10^–15^ M (Supporting Information). Minute volumes of 1.0 M KBr were then added to the SE(R)RS samples to a final concentration of 5.0 × 10^–2^ M. KBr is known to induce the formation of AgNP “hot-spots” and to increase the spectral reproducibility.^46,47^ Br^–^ anions can surround AgNPs and facilitate the formation of strong electrostatic bridges with the R6G^+^ cations in aqueous colloids near neutral pH. Samples were allowed to incubate for 24 h to maximize R6G adsorption to AgNPs, and spectra were collected in 2 mL quartz cuvettes. The quartz cuvettes were precleaned using a two-step procedure. In the first step, the cuvettes were rinsed with ethanol and then heated at 500 °C for 24 h. In the second step, the cuvettes were placed in an aqua regia solution for 1 h, thoroughly rinsed with HQ H_2_O for 1 h, and then stored in ethanol until use.

Raman and SE(R)RS measurements (Figures S3–S6) were performed on a confocal LabRam HR 800 system (Horiba, Inc.) using a continuous-wave internal HeNe laser (632.8 nm, 22 mW, Raman and SERS) and a frequency-doubled-diode-pumped Nd/YAG laser (Laser Quantum, 532.1 nm, 15 mW, SERRS). The confocal hole was set at 300 μm, and a holographic grating of 600 grooves mm^–1^ was selected. Raman scattered photons were collected in a backscattering geometry through an optically coupled 50× microscope objective and a thermoelectrically cooled, deep-depletion back-illuminated Andor CCD camera (1024 × 256 pixels). The spectral resolution was ∼1 cm^–1^. Substrate “blanks” (i.e., SE(R)RS samples containing only AgNPs and KBr but no R6G) were analyzed in between each SERRS and SM-SERRS sample to ensure there was no crossover R6G.

### 2.8 Fluorescence Emission Spectroscopy of R6G

The fluorescence emission spectra of 10^–6^–10^–8^ M R6G aqueous solutions were recorded in the presence and absence of AgNPs (Figure S7 and Table S3). The fluorescence emission of R6G was stimulated at 530 nm, and the magnitude of photon emission was quantified at 550 nm using a Cary Eclipse Fluorescence spectrophotometer (Agilent Technologies) at a scan rate of 2400 nm min^–1^. The acquisition parameters were: PMT voltages of 600, 750, and 900 V. The spectral resolution was of ∼1 nm.

### 2.9 Data Processing

Spectral data processing, figure preparations, and statistical analysis were carried out in the LabSpec 6, Origin Pro, the ImageJ 1.46R, ChemDraw, and BioRender software. Original spectra were baseline-corrected using a B-spline function, and spectral features of interest such as peak position, full width at half-maximum (fwhm), intensity, and integrated area were established.

### 2.10 FDTD Simulations for SE(R)RS

ANSYS Lumerical FDTD was used to model and analyze the behavior of electromagnetic fields (EMFs) in assemblies of one, two, and three spherical AgNPs of 50 nm in diameter.^1^ The geometry and complex dielectric function of the nanospheres were defined within a sufficiently large simulation domain with perfectly matched layer boundaries. A plane wave source covered the relevant wavelength range. The spatial grid resolution and time step were chosen to ensure the numerical stability and accuracy. The electric and magnetic fields were recorded to compute absorption, scattering, and extinction cross-sections. Near-field and far-field analyses were performed to examine local field enhancements and scattering patterns. Simulations were conducted with convergence verified through grid refinement.

## 3 Results and Discussion

### 3.1 TFF-Based Fractionation and Physicochemical Characterization of AgNPs

Three independent batches of original Creighton colloid (ORI) were successfully fractionated (AgNP ≥ 40 and AgNP ≤ 40), and the physicochemical properties of AgNPs governing the SE(R)RS enhancements were characterized.

#### 3.1.1 AgNP Size, Shape, and LSPR

**Figure 2:**
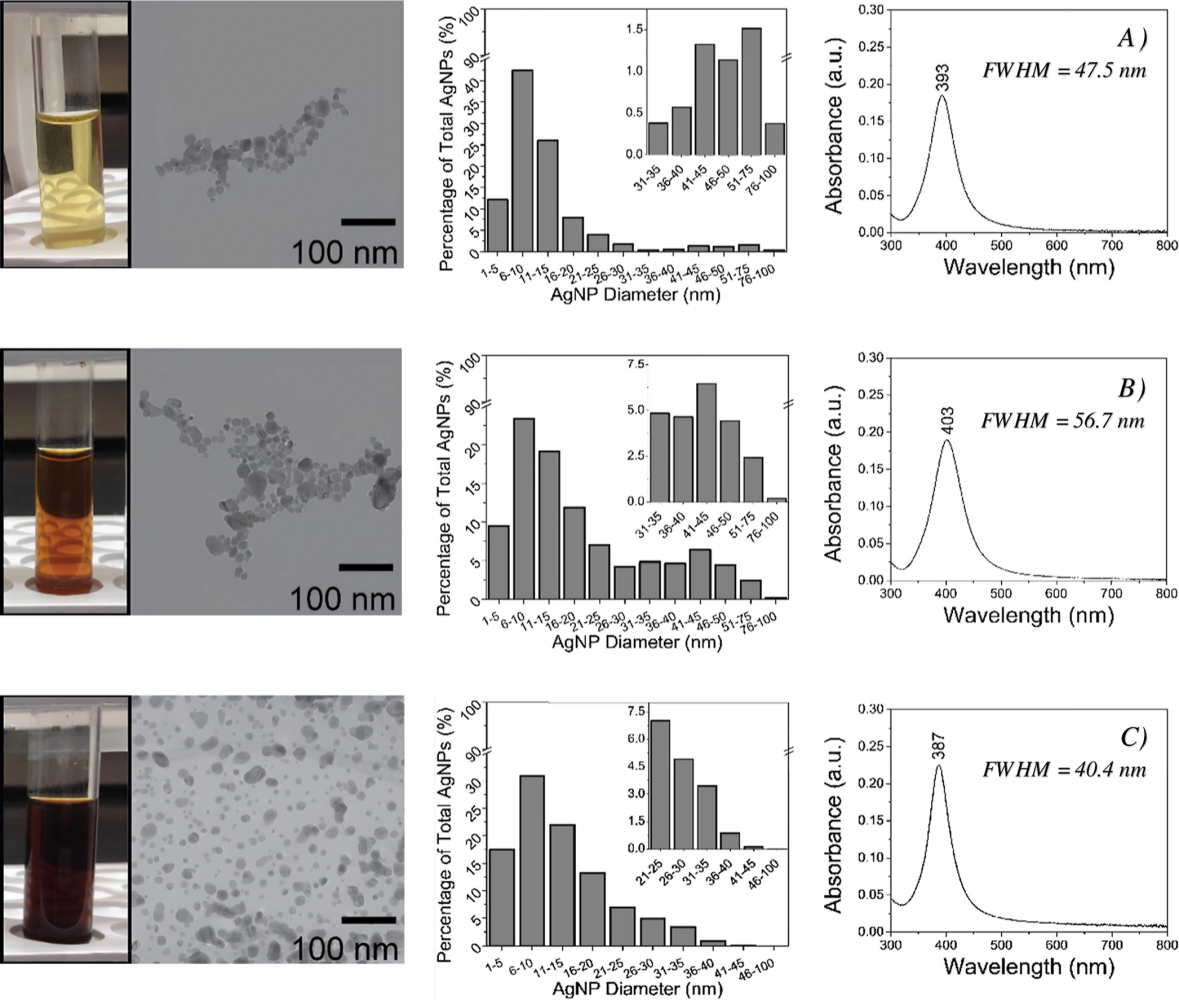
Physicochemical characterization of (A) the original Creighton colloid (ORI) of AgNPs, and the two TFF fractions for SE(R)RS, namely, (B) AgNP ≥ 40 and (C) AgNP ≤ 40. Following data is presented from left to right in each row: visual images, TEM micrographs, TEM size histograms (*n* = 500 AgNPs), and UV–vis absorption spectra of each colloidal sample.

The TEM data showed that ORI (Figure 2A) was mostly represented by quasi-spherical AgNPs of moderate size distribution in the 1–100 nm range (Table S2). These Creighton AgNPs are negatively charged (−28 ± 2 mV at pH of 6.8) and smaller in size (mostly ≤ 40 nm) than the light source wavelength and thus experience negligible scattering. Scattering and radiation damping effects dominate the optical response of AgNPs of ∼100 nm in diameter and larger.^47−49^ Following the Mie theory, the total extinction of ORI depends on only the light absorbance. The UV–vis absorption spectrum of ORI exhibited a single, sharp, Gaussian-shaped LSPR maximum at 393 nm (Figure 2A), which supports the TEM observations of round-shaped AgNPs of low aggregation. It should be noted that the number of LSPR peaks in an extinction spectrum decreases as the symmetry of AgNPs increases.^46−49^ Small AgNP spheres give rise to only one plasmonic resonance (i.e., one LSPR mode), which is independent of the direction of the incident light. The two TFF fractions, AgNP ≥ 40 (Figure 2B) and AgNP ≤ 40 (Figure 2C), retained the single LSPR peak but experienced considerable changes in their spectral profile. The LSPR position shifted from 393 nm (ORI) to 403 nm (AgNP ≥ 40) and 387 nm (AgNP ≤ 40), indicating an increase and a decrease, respectively, in the size of AgNPs in the two fractions.

The TEM measurements confirmed these trends (Figures 2B,C): TFF successfully isolated the large AgNPs or AgNP-aggregates (≥ 40 nm) in the 50 nm-retentate. In this process, the average diameter of AgNPs increased from 13.4 nm (ORI) to about 20.1 nm (AgNP ≥ 40), and the FWHM increased by 9.2 nm after the 50 nm-filtering. This was expected because the LSPR bands become wider, less symmetric, and red-shifted with the increase in the size of AgNPs because of surface dispersion effects.^45−49^ This means that the dipolar-induced modes are accompanied by other higher multipolar charge distributions as AgNPs aggregate or become less symmetric at large sizes (≥ 40 nm).^49^ An opposite absorption trend was noticed for AgNP ≤ 40, where the LSPR peak position was left-shifted to 387 nm and became narrower with a FWHM of 7.1 nm (Figure 2C). Accordingly, AgNP ≤ 40 is made of small, homogeneous AgNPs of an average diameter of 12.4 nm, low aggregation, and narrower size distribution than ORI. The size-selection process was further confirmed by TEM data showing maximum AgNP diameters of ∼41 and ∼77 nm in AgNP ≤ 40 and AgNP ≥ 40, respectively (Table S2).

#### 3.1.2 AgNP Concentration, Purity, and Environment

Visual inspections (Figure 1) along with ICP-OES quantifications (Tables S1 and S2) offered corroborating data that the TFF procedure was also successful in concentrating and purifying the original colloid of AgNPs. A change in color from golden yellow for ORI to orange brown for AgNP ≥ 40 and finally dark brown for AgNP ≤ 40 was indicative of the concentration degree of AgNPs. ICP-OES showed that the AgNP concentrations were ∼10- and 90-fold higher in AgNP ≥ 40 and AgNP ≤ 40, respectively, than in ORI. The two TFF retentates had a considerable shelf life (a few months) and showed no significant changes in optical properties (lasting LSPR profiles). Significantly higher TFF concentration degrees (over 550-fold and ∼8500 μg mL^–1^ of total Ag) were also obtained, but with lower AgNP stability and accelerated filter fouling.^35−37^ Furthermore, the TEM micrographs and size histograms confirmed that the TFF procedure was successful in increasing the concentration of AgNPs without inducing significant AgNP flocculation (Figure 2).

The TFF manipulation was also successful in removing most of the excess reagents, byproducts, and solvent. For example, the volume of water carrying excess reagents and byproducts was significantly reduced during the TFF (e.g., from 3.85 L for ORI down to 10 mL for AgNP ≤ 40). This is the most commonly used solvent in the bottom-up fabrication of colloidal AgNPs. The purification was confirmed through the ICP-OES quantification of Na from NaBH_4_, the reducing agent in the redox reaction of Ag^+^ from AgNO_3_, which results in the formation of AgNPs. Significant decreases in Na content (e.g., ∼54–62%) were observed in both TFF fractions collected for SE(R)RS experiments (Table S1). Further purification evidence was brought by the Raman measurements of the colloidal samples of AgNPs before and after TFF. As illustrated below, a few Raman bands associated with post synthetic species present in the colloidal matrix (e.g., nitrate ions) were significantly reduced through TFF.

The purity of a SE(R)RS substrate is of extreme importance for the quantitative interpretation of molecular spectra. “Anomalous” Raman bands are a disadvantageous artifact in most colloidal preparations, including the Creighton colloid,^50−52^ and carry the potential to interfere with Raman vibrational bands of the molecule(s) of interest. Additionally, the LSPR is dependent on the local dielectric environment.^49,53^ For example, the LSPRs in a medium of refraction index *n* > 1 are red-shifted with respect to those in a vacuum. Thus, impurities and ionic byproducts within the colloidal matrix can have undesirable effects on the EM enhancement during SE(R)RS, such as increases in the refraction index (*n* = 1.33 for water at 20 °C and VIS light). Salts such as KBr are typically added for promoting the formation of SE(R)RS “hot spots” and for enhancing the Raman signal of potential anomalous bands by promoting the self-assembly of AgNPs.^50−52^ In the presence of only KBr, ORI exhibited a few Raman bands (Figure S3) associated with post synthetic species present in the colloidal matrix (e.g., nitrate ions and inorganic ions^50−52^). After the TFF processing of ORI, the intensity of these Raman bands was significantly decreased in AgNP ≤ 40 (by ∼70%) when compared to the characteristic vibrational modes of water (e.g., stretching mode at ∼1640 cm^–1^) of constant concentration. The decreased intensity of “anomalous” Raman bands, along with the decrease in Na content is the result of TFF processing. Thus, TFF not only aids in the purification of colloidal AgNP systems but also helps eliminate or significantly reduce spectral interferences associated with byproducts or excess reagents depending on the selected level of volume reduction.

Overall, the size-selection, concentration, and purification of unfunctionalized, quasi-spherical AgNPs were completed within the same TFF setup. This ecofriendly manipulation of AgNPs for SE(R)RS saved time (∼completed within a few hours), reduced costs (reusing filters), and minimized safety risks (fully integrated systems with set-point controls). The resulting TFF fractions exhibited enhanced purity (reduced excess reagents and byproducts), high but stable concentrations (∼100-fold larger than in ORI for months), and a clear size separation of small AgNPs (AgNP ≤ 40) and large AgNPs (AgNP ≥ 40), with expected SE(R)RS benefits of increased sensitivity and reproducibility. However, the TFF procedure requires further improvements to increase yields and reduce size deviations in the separation process. Herein, the AgNP ≥ 40 fraction also contained small AgNPs (<40 nm), probably due to membrane fouling or damages associated with the high chemical affinity of the aqueous AgNPs to the exposed thiol groups of the polysulfone membranes, as well as the repeated filter usage and acid cleaning.^37^ Future studies can explore using nonreusable filters or tailored membrane filters with hydrophilicity and antifouling to eliminate these limitations. These improvements can be at the expense of increased experimental durations and costs.^54,55^ Furthermore, filters of increasingly larger or smaller sizes (e.g.,1–1000 kDa) can be sequentially employed to further purify and fractionate AgNPs into narrow size distributions as needed for SE(R)RS.

### 3.2 SERS under the Pre-Resonance Excitation of R6G (10^–6^–10^–9^ M) at 632.8 nm

It has been previously shown that the SERS signal of R6G adsorbed on various colloidal AgNPs saturates at R6G/AgNP molar ratios of 3:1 or greater.^12,56^ Herein, R6G concentrations of 1 × 10^–6^–10^–15^ M were selected for testing the SERS-based sensing capabilities of ORI and the two TFF fractions. The R6G/AgNP number ratios of this study corresponded to subunit values (1.2 × 10^–2^ to 1.2 × 10^–4^ ratios—Table S4) that can facilitate the accurate estimations of the SE(R)RS EFs at submonolayer coverages of AgNPs with R6G. It should be noted that the R6G dye solution has absorption and fluorescence emission maxima at ∼528 and ∼550 nm, respectively.^35,47^ Thus, the concentrations of aqueous R6G solutions for SE(R)RS were spectrophotometrically confirmed via the 528 nm absorption maximum (π*−π transitions), while the adsorption of R6G onto AgNPs was quantified via the reduction in the fluorescence emission of R6G at 550 nm (Supporting Information calculations; Table S3). It is well-known that the fluorescence of the R6G dye is quenched via metal complexation.^2,35^ The two TFF fractions (≥91%) were found to adsorb R6G more effectively than ORI (≥78%) at all R6G concentrations.

**Figure 3:**
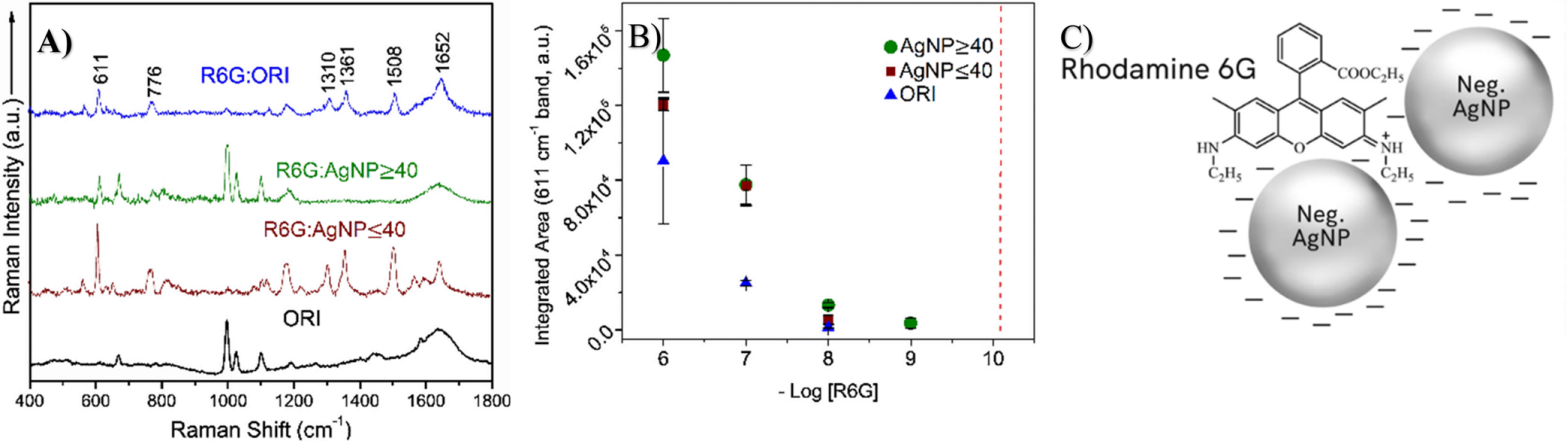
(A) Baseline-corrected SERS spectra of rhodamine 6G (R6G) incubated with original Creighton colloid, ORI, and the two TFF fractions of colloidal AgNPs: AgNP ≥ 40 and AgNP ≤ 40. R6G concentration in each spectrum is 10^–8^ M, except for the R6G/AgNP ≥ 40 sample of 10^–9^ M. The spectra were intensity shifted for clarity and the Raman spectrum of ORI (no R6G) with anomalous bands was provided as a control (no *Y* axis offset). (B) Plot of the integrated area of the 611 cm^–1^ peak of R6G versus the negative log of the R6G concentration (mol L^–1^). Error bars represent the standard deviations of *n* = 3 independent experiments, while the red dash marks the concentration of R6G at which single-molecule (SM) detection events would occur based on laser focal volume (F.V.) calculations (Supporting Information, calculations). (C) Possible adsorption of R6G cations at the “hot-spot” between two electrostatically bridged AgNPs of negative surface charge (objects are out-of-scale for illustrative purposes).

SERS spectra of R6G incubated with AgNPs from ORI, AgNP ≥ 40, and AgNP ≤ 40 had vibrationally rich signatures down to 10^–8^ M (Figures 3A and S3).

#### 3.2.1 Size-Dependence of AgNPs

However, only AgNP ≥ 40 enabled the detection of R6G at 10^–9^ M (Figures 3B and S3) and none of the three colloidal samples reached the SM detection regime under pre-resonance excitation at 632.8 nm. The increase in both the detection limit (10^–9^ M) and the integrated area of the 611 cm^–1^ marker band of R6G (in-plane C–C–C ring vibration)^57^ is probably the result of the greater EM enhancement associated with the larger size of AgNPs in AgNP ≥ 40 (maximum diameter of ∼77 nm) when compared to AgNP ≤ 40 (maximum diameter of ∼41 nm). Prior experimental work^29^ showed a linear relationship between the size of spherical AgNPs and the SERS intensity of R6G molecules that were excited with an off-resonance laser at 785 nm. Herein, the observed AgNP size-dependence of the SERS signal was observed despite the lower Ag content of AgNP ≥ 40 (106.8 μg mL^–1^) when compared to AgNP ≤ 40 (899.9 μg mL^–1^). These Ag concentrations correspond to a total number of ∼4.8 × 10^12^ and ∼1.7 × 10^14^ AgNPs in the SE(R)RS colloidal volume (2 mL) of AgNP ≥ 40 and AgNP ≤ 40, respectively (Table S4).

#### 3.2.2 Concentration-Dependence of AgNPs

The two TFF fractions were diluted to the Ag concentration of ORI (10.1 μg mL^–1^) for further comparisons. As evident from Figure S8, the low detection limit of AgNP ≥ 40 and the higher SERS intensity of the 611 cm^–1^ band were retained even at a smaller Ag content as in ORI (∼10-fold decrease to 10.1 μg mL^–1^ of Ag or 1.6 × 10^12^ of AgNPs in 2 mL). This further supports our hypothesis that the SERS enhancements under off-resonance or pre-resonance conditions for R6G will increase with the size of the quasi-spherical AgNPs.

Overall, these SERS experiments demonstrate that the size of AgNPs is one of the most important parameters governing the SERS effect in off-resonance or pre-resonance excitation of R6G. It should be noted that the pre-resonance contributions of R6G at 632.8 nm are considered negligible.^58^ The largest SERS intensities for R6G were obtained using the TFF fraction containing most of the Creighton AgNPs of ∼40–50 nm diameter, i.e., AgNP ≥ 40. However, the high Ag concentration of small AgNP ≤ 40 (899.9 μg mL^–1^) can compensate for the difference in AgNP size when compared to large AgNP ≥ 40 (106.8 μg mL^–1^) and lead to additional enhancements at high analyte concentrations (e.g., 10^–7^ and 10^–8^ M of R6G—Figure 3B). This is probably due to the higher number of small, homogeneous AgNP ≤ 40 that are present within the F.V., allowing for effective adsorption of R6G molecules. The total AgNP SA available for R6G adsorption in the colloidal samples of AgNP ≥ 40 and AgNP ≤ 40 (2 mL cuvette) was estimated to be 6.1 × 10^–3^ m^2^ and 2.50 m^2^, respectively, using geometric arguments (AgNPs of aspect ratios of 1.0–1.2) and the observed TEM diameters (Table S5).

### 3.3 SERRS under the Resonance Excitation of R6G (10^–8^–10^–15^ M) at 532.1 nm

**Figure 4:**
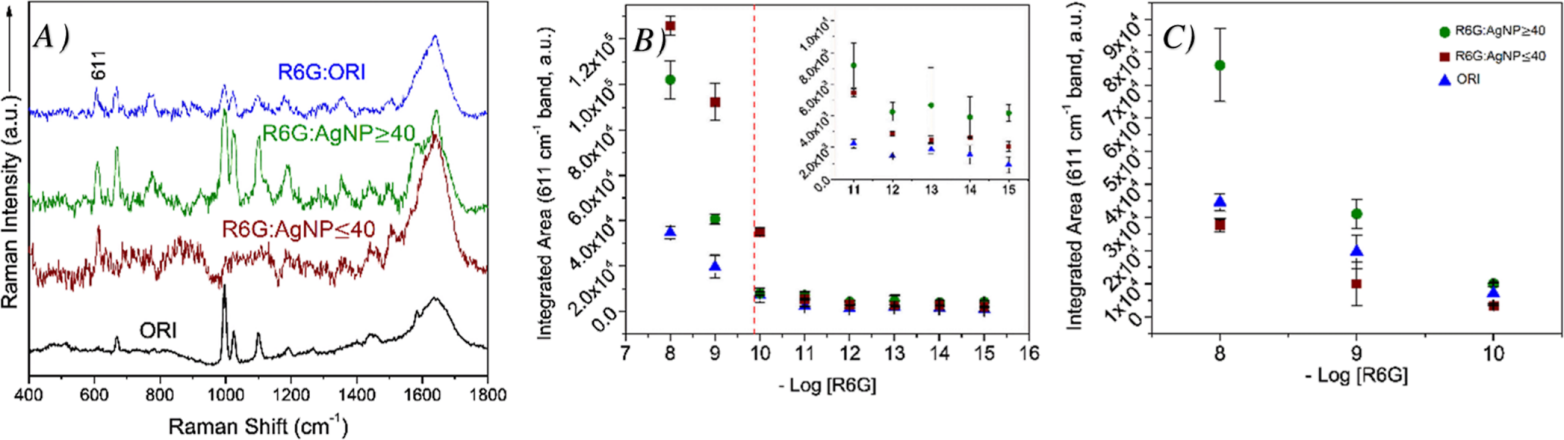
(A) Baseline-corrected SERRS spectra of 10^–15^ M of R6G incubated with ORI and the two TFF fractions of colloidal AgNPs: AgNP ≥ 40 and AgNP ≤ 40. The spectra were intensity shifted for clarity. Same intensity scale applies to each spectrum aside from the Raman spectrum of ORI control (no R6G). Plot of (B) the integrated area of the 611 cm^–1^ peak of R6G versus the negative log of the R6G concentration (mol L^–1^), and (C) the values of the same marker band of R6G adsorbed onto AgNP ≥ 40 and AgNP ≤ 40, after being diluted down to a Ag content equal to ORI (10.1 μg mL^–1^). Inset in (B) shows the expanded region for the SM regime. Error bars represent the standard deviations of *n* = 3-independent TFF experiments, while the red dash marks the concentration of R6G at which SM detection events would occur based on laser F.V. calculations (Supporting Information, calculations).

At R6G concentrations ≥10^–8^ M, large fluorescence backgrounds were observed for all colloidal samples even after 48 h of incubation time with AgNPs. This is due to the larger fluorescence scattering cross-section of R6G when compared to its Raman scattering cross-section at 532.1 nm excitation. At R6G concentrations <10^–8^ M, the SERRS signal was largely unaffected by the fluorescence of R6G, and vibrationally detailed spectra could be obtained for all colloidal substrates (ORI, AgNP ≥ 40, and AgNP ≤ 40) over several orders of magnitude (10^–8^–10^–15^ M of R6G—Figures S5 and S6). Furthermore, the resonant SERRS spectra at 532.1 nm exhibited significantly higher intensities and reproducibility (Figure 4—90–93%) when compared to the pre-resonant SERS spectra of similar R6G concentrations at 632.8 nm (Figure 3—67–92.3%). This is mainly due to additional RE contributions associated with the proximity of the laser line (532.1 nm) to the electronic transition of the R6G molecule (528 nm). Previous SERRS studies also indicated the RE mechanism can be on the order of or larger than the EM mechanism.^9,12,15^

#### 3.3.1 Above and Near the Single-Molecule Threshold of R6G (10^–8^–10^–10^ M)

The magnitude of the RE enhancement for AgNP ≤ 40 at 532.1 nm is nearly the same as or larger than the EM enhancement of AgNP ≥ 40 at 632.8 nm. Figure 4B shows that the integrated areas of the 611 cm^–1^ marker band were higher for AgNP ≤ 40 than for AgNP ≥ 40 at all R6G concentrations above the SM threshold (Figure 4B). The presence of ∼36-fold more AgNPs in the F.V. of AgNP ≤ 40 than in AgNP ≥ 40 (Table S6) results in more R6G molecules experiencing resonance excitation and subsequent Stokes emissions at this laser excitation. Herein, the F.V. was estimated to be 1.13 × 10^–14^ L at 532.1 nm based on the optical diffraction theory (eq 1)
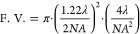
1where λ is the excitation wavelength and *NA* is the numerical aperture of the objective lens (Supporting Information, calculations).

The considerable RE contributions were further supported through differential fluorescence measurements of AgNP ≤ 40 before and after incubation with R6G. AgNP ≤ 40 experienced the largest degrees of R6G adsorption onto AgNPs and subsequent fluorescence quenching (Table S3). If the hypothesis holds, then a significant decrease in SERRS intensities should be observed for AgNP ≤ 40 at a Ag concentration equal to that of ORI (10.1 μg mL^–1^). Indeed, a nearly 10-fold reduction in the integrated area of the 611 cm^–1^ mode was noticed at 10^–8^ and 10^–9^ M of R6G (Figure 4C), when the Ag content of AgNP ≤ 40 was diluted to that of ORI. Furthermore, the highest SERRS signals were measured for the diluted AgNP ≥ 40 (10.1 μg mL^–1^). These spectral changes reflect the additional EM contributions brought by the larger size of the diluted AgNP ≥ 40 and the narrower gap between their LSPR (403 nm) and the resonant laser line of R6G (532.1 nm).^41^

#### 3.3.2 Below the SM Threshold of R6G (10^–11^–10^–15^ M)

It is well-known that the structure–function relationship of AgNPs and adsorbed species is best examined at the SM or near-SM level.^59,60^ Therefore, additional SM-SERRS measurements were performed at ultralow concentrations of R6G to ensure a favorable SM regime (Figures 4, S5, and S6). The lowest SM-SERRS detection limit was established at 10^–15^ M (Figure 4A), and several scans were averaged together for spectral evaluations (Figure 4). The “blinking” behavior characteristic of the SM regime was observed for minute droplets of R6G onto a glass microscope slide until the water dried out completely. There is statistically about one R6G molecule present within the F.V. at a R6G concentration of 1.0–5.0 × 10^–10^ M under both laser excitation conditions (Supporting Information, calculations—Table S6). As postulated, AgNP ≥ 40 resulted in higher SM-SERRS signals than AgNP ≤ 40 or ORI below the SM threshold (Figure 4B), where the EM enhancement arising from the size-dependence of the large AgNPs prevailed. However, the RE enhancement was also considerable. The integrated areas of the 611 cm^–1^ band for AgNP ≤ 40 followed those for AgNP ≥ 40 (Figure 4B). Furthermore, only a small percentage of the observed AgNPs in the diluted fraction of AgNP ≥ 40 were represented by dimers or other small aggregates close to or below the SM regime (Figure S9).

Overall, there was a size-dependence of AgNPs on the SM-SERRS effect under the resonance excitation of R6G at 532.1 nm. Above or near the SM level (10^–8^–10^–10^ M of R6G), the largest signals were detected for the small but highly concentrated AgNP ≤ 40. However, comparable or larger signals were obtained above or near the SM for AgNP ≥ 40, after the TFF fractions were both diluted to the metal concentration of ORI. Below the SM threshold (10^–11^–10^–15^ M of R6G), the SM-SERRS regime was favored by the large AgNP ≥ 40.

### 3.4 SE(R)RS EFs

The extensive characterization of the Raman spectrum of R6G has resulted in its routine use as a molecular probe for the determination of overall SE(R)RS EFs arising from different nanostructured substrates.^2,4,9,12,15,35,47,61^ Although many different forms of SE(R)RS EFs have been reported in the literature, the surface EF (SEF) rigorously determines the total signal enhancement on a near per-molecule basis for adsorbed species such as R6G.^43^ Moreover, the SEF (eq 2) considers the geometric properties of the nanosubstrate and the fact that only adsorbed species experience enhanced Raman scattering
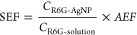
2where *C* is the concentration of R6G adsorbed onto AgNPs or in a bulk solution (no AgNPs) and analytical EF (AEF) is the AEF. The R6G concentration before and after adsorption of AgNPs was determined through fluorescence emission measurements (Table S3). While most studies refer to the AEF as the simple ratio of the SE(R)RS intensity to the regular Raman intensity of a particular vibrational mode, it is important to note that increases in the intensity of specific vibrational modes can be due to a concurrent increase in the scattering cross-section (σ) of the vibrational coordinates. Hence, a more thorough estimation of the AEF and SEF must consider the increase in the vibrational scattering cross-section.^8,35,47^ AEF (eq 3) can be calculated as
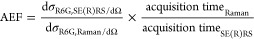
3where dσ_R6G,SE(R)RS/dΩ_(eq 4) is the differential scattering cross-section for a particular vibrational mode.
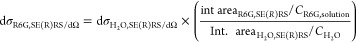
4where int.area is the integrated area of the selected marker band in the SE(R)RS spectrum of the analyte. Thus, the choice of a particular vibrational mode is crucial for correctly understanding the EFs reported for nanostructured substrates in SE(R)RS. In this study, the well separated Raman marker, intense bands of R6G at 611 cm^–1^ (in-plane vibration of the xanthine backbone), and water at 3241 and 3394 cm^–1^ (symmetric and asymmetric stretching modes) were selected for the determination of the EFs.^35,47,57,62^ It should be noted that the water concentration was kept constant throughout the experiments (55.5 M).

**Figure 5:**
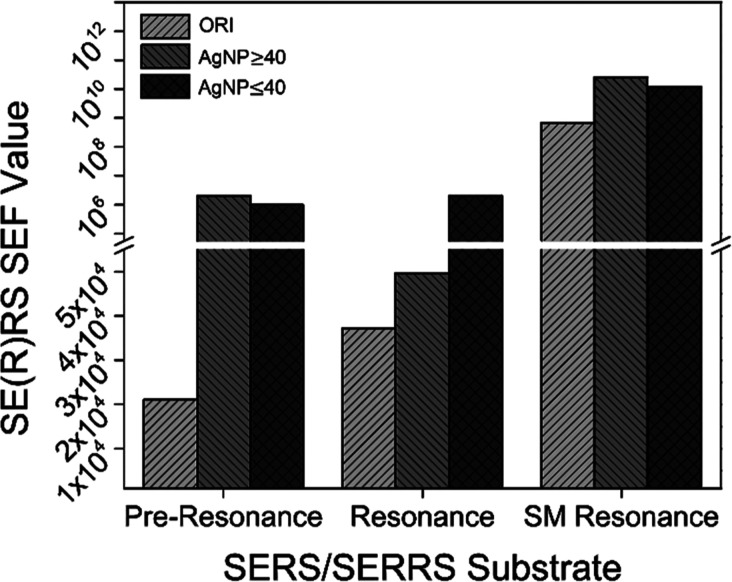
Calculated surface EF (SEF) values for the original colloid (ORI) and the two TFF fractions, AgNP ≥ 40 and AgNP ≤ 40, under pre-resonance (10^–8^ M of R6G), resonance (10^–9^ M), and SM resonance excitation conditions for R6G (10^–15^ M).

The SEF values of the two TFF fractions were markedly superior to those of ORI under all excitation conditions for R6G (Figure 5).

#### 3.4.1 SERS

Under pre-resonance conditions at 632.8 nm, the larger and more concentrated AgNP ≥ 40 resulted in the largest, ∼100-fold increase in SEF (2.1 × 10^6^) of R6G (10^–8^ M) when compared to ORI (2.0 × 10^4^). Evidently, both the size and the concentration of AgNPs play a key role in the observed enhancements with substantial EM contributions. However, the larger number of AgNPs (Table S4) available in the SE(R)RS cuvettes (2 mL) of AgNP ≤ 40 (1.7 × 10^14^) than AgNP ≥ 40 (4.8 × 10^12^) and ORI (1.6 × 10^12^) can compensate for the size-dependence of the EM mechanism and leads to comparable SEF values at relatively higher R6G concentrations (e.g., 10^–8^ M).

#### 3.4.2 SE(R)RS

Under resonance conditions at 532.1 nm, AgNP ≤ 40 led to the greatest SEF value (2.0 × 10^6^) through RE contributions and an increase in the Raman scattering cross-section of R6G. Prior studies with a resonance laser quatiat 514 nm demonstrated that a modest SEF of 10^4^ can result in a significant increase in the Raman scattering cross-section of R6G (from 10^–24^ to 10^–20^ cm^2^ sr^–1^) and subsequent SM detection events.^2,63^

#### 3.4.3 SM-SE(R)RS

Under SM resonance conditions at 532.1 nm, in which the AgNP concentration plays a minute role, AgNP ≥ 40 gives again rise to the largest SEF values (2.5 × 10^10^). It should be noted that all three nanosubstrates (ORI, AgNP ≥ 40, and AgNP ≤ 40) led to SEF values (6.7 × 10^8^, 2.5 × 10^10^, and 1.2 × 10^10^, respectively) close to or above the values reported in the literature for SM detection events (SEF of ∼10^8^–10^10^).^2,9,12,58^ These SEF values are realistic; larger values of 10^14^–10^15^ were recently acknowledged as “artificially inflated”^2^ because of the misleading use of nonresonant cross-sections for resonant analytes such as R6G.

Finally, it should be noted that CE contributions have not been included in this discussion, but they could add a molecule-dependent SEF value of 10^1^–10^2^ to the EM and RE contributions to SEF.^2,58^ In the colloidal samples, R6G exists as a cation that can form electrostatic bridges with the negatively charged AgNPs and thereby position itself within “hot-spots” (AgNP-R6G-AgNP, Figure S4C). This adsorption geometry of immediate vicinity to AgNPs led to the observed fluorescence quenching of R6G, which typically occurs through an electron transfer mechanism.

### 3.5 FDTD in SE(R)RS

**Figure 6:**
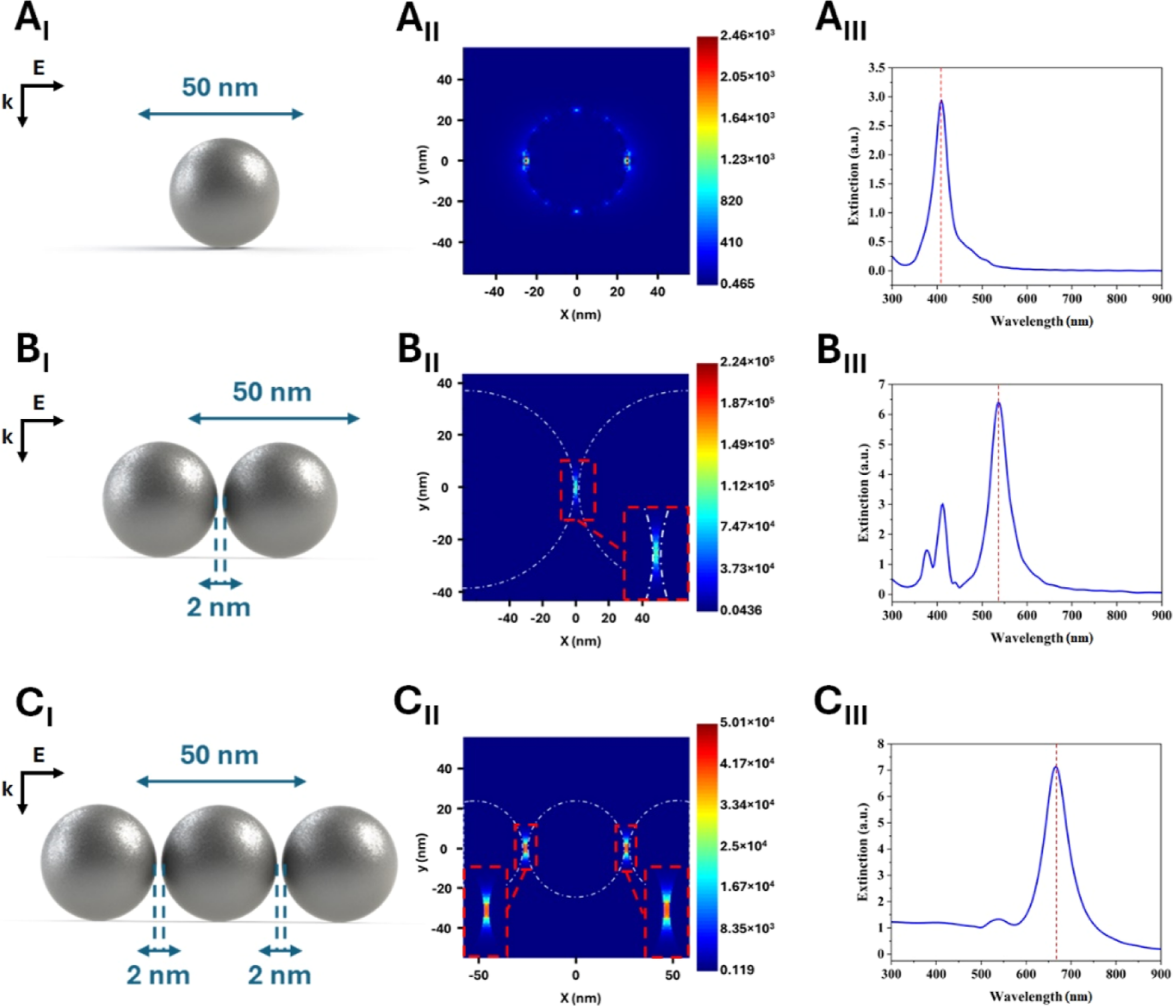
Schematic showing: (left column) 3-D geometry of a single (A_I_–A_III_), dimer (B_I_–B_III_), and trimer (C_I_–C_III_) of spherical AgNPs of 50 nm in diameter with a minimal separation of 2 nm, (middle column) the plasmonic electromagnetic field contours and the corresponding enhancement values around the AgNPs, and (right column) the calculated extinction spectra showing a redshift in the plasmonic maximum upon increasing the number of AgNPs in the system. Incident light is polarized along the interparticle axis (A_I_, B_I_, and C_I_). Propagation and polarization axes are shown.

FDTD is one of the most powerful methods for performing electrodynamic calculations of light scattering from NPs of arbitrary shapes and sizes. FDTD simulates the distribution of the EMF around the irradiated NPs by numerically solving Maxwell’s equations.^64−70^ Here, FDTD was utilized to examine the role of plasmonic field coupling in assembled spherical AgNPs of 50 nm diameter (single, dimer, and trimer) and their SE(R)RS-based detection efficiency. The most favorable conditions for SE(R)RS with the incident light being polarized along the interparticle axis were selected (Figure 6—A_I_, B_I_, and C_I_). At very narrow gap sizes of ∼2 nm (R6G monomer is ∼1.6 nm), the plasmonic coupling interaction can be approximated as the interaction between dipoles on each AgNP. The plasmonic maximum of AgNPs experienced a redshift from 400 to 665 nm as the number of AgNPs in the system increased from one to three (Figure 6—A_III_, B_III_, and C_III_). This suggests that there is a strong dipolar coupling between the plasmonic AgNPs, and this is enhanced with the increase in number of particles. Hence, high EMFs or “hot spots” along the 2 nm separation distance are created (Figure 6—B_III_ and C_III_). This behavior was also reported in the literature for a dimer of spherical AgNPs of 36 nm in diameter and 2 nm separation.^64^ Other potential interactions between multipolar modes of individual AgNPs can also arise and lead to additional enhancements under favorable laser excitations (Figure 6, B_III_ and C_III_). For example, three distinct plasmonic modes were observed for the AgNP–AgNP dimer at the interparticle distance of 2 nm. As one would expect, the largest enhancements were detected at the “hot spot” of AgNP dimers and trimers when compared to single AgNPs (Figure 6—A_II_, B_II_, and C_II_). These dipole resonances at the interfacial region between AgNPs correspond to the strongest extinction maxima at ∼540 and 665 nm, which come very closely to the laser excitations in the SE(R)RS experiments at 532.1 nm (resonance) and 632.8 nm (pre-resonance for R6G in aqueous solution), respectively. Thus, the results of the FDTD simulations support the proposed hypothesis and further confirm the interpretation of the experimental SER(R)RS data. More specifically, the largest enhancements and SM-SERRS detection events were experimentally detected with a 532.1 nm laser for the TFF fraction (AgNP ≥ 40) containing most AgNPs of 40–50 nm in diameter. Under these experimental resonance conditions (532.1 nm), R6G cations were strongly adsorbed onto AgNPs, and probably self-assembled into dimers or trimers through AgNP^–^-R6G^+^-AgNP^–^ electrostatic bridges (Figure 3C). The FDTD simulations indicate that large SERS enhancements can be created under off-resonance or pre-resonance conditions (632.8 nm) through the formation of trimers of large AgNPs (∼40–50 nm in diameter). This can probably occur at relatively high concentrations of R6G (10^–6^–10^–9^ M) and for a larger number of AgNPs available within the F.V. (46 AgNPs for AgNP ≥ 40 versus 15 AgNPs for Ori—Table S6). Thus, TFF could facilitate these additional enhancements through the rapid concentration of large AgNPs in AgNP ≥ 40.

## 4 Conclusions

This study demonstrated that TFF can facilitate the ecofriendly isolation of Creighton AgNPs of controlled morphology (size, concentration, and purity) for enhancing their SE(R)RS-based sensing capabilities. The membrane-based separation of spherical AgNPs with a cutoff diameter of ∼40 nm was completed within a few hours and without the use of additional solvents or hazardous reagents. The TFF fractions of moderate Ag concentrations (e.g., AgNP ≥ 40) were stable for months (i.e., plasmonic properties) and were largely void of excess reagents and byproducts (e.g., AgNP ≤ 40). Overall, the proposed hypotheses related to the two TFF fractions, AgNP ≤ 40 and AgNP ≥ 40, were supported by both experimental (SE(R)RS) and theoretical results (FDTD). More specifically, the SEF of the two resulting TFF fractions were markedly superior to those of the original colloid under all excitation conditions, and highly reproducible (>90% in SERRS) signals were obtained. Furthermore, both TFF isolates were more effective in adsorbing the R6G analyte (≥91%) than the original colloid (≥78%) at submonolayer coverages. SERS under pre-resonance excitation at 632.8 nm: only AgNP ≥ 40 enabled the detection of R6G at 10^–9^ M and produced the largest SEF (2.1 × 10^6^). SERRS and SM-SERS under resonance excitation at 532.1 nm: below the SM threshold, AgNP ≥ 40 gave rise to the largest SM-SEF values (2.5 × 10^10^) down to 1 × 10^–15^ M of R6G. Above or near the SM threshold, AgNP ≥ 40 had larger SEF values than AgNP ≤ 40 at comparable Ag concentrations. Nevertheless, AgNP ≤ 40 compensated for the size-dependence of the EM enhancements at larger TFF Ag concentrations, which led to comparable SEF values to AgNP ≥ 40 via additional REs. TFF resulted into a 100-fold increase (AgNP ≤ 40 versus ORI) in the number of negatively charged AgNPs that were available to electrostatically bridge R6G cations and form SERRS “hot-spots” (AgNP-R6G-AgNP) within the F.V. In conclusion, TFF is an ecofriendly, time- and cost-effective separation process that could be applied to a large variety of spherical, colloidal models of plasmonic NPs. TFF could eliminate the need to functionalize colloidal NPs for controlling their morphological or plasmonic properties. Capping and functional reagents can be hazardous, exhibit reversible affinity to NPs, or block target analytes from attaching to the NP surface. Filters of large or small sizes (1–1000 kDa), different SAs (13 cm^2^–11.35 m^2^), or tailored properties (hydrophilicity and antifouling) can be sequentially employed in TFF to further fractionate NPs at both research lab and industry scale for reproducible and stable SE(R)RS.

## Supporting Information Available

TFF system, physicochemical properties of AgNPs, and their SERS-based sensing capabilities (PDF)

## Abbreviations

AEF: analytical enhancement factor
AgNPs: silver nanoparticles.
AgNP ≥ 40: silver nanoparticles of 40 nm diameter and larger in the 50 nm-retentate
AgNP ≤ 40: silver nanoparticles of 40 nm diameter and smaller in the final 30 kDa-retentate
EF: enhancement factor
FDTD: finite-difference time-domain
LSPR: localized surface plasmon resonance
ORI: original Creighton colloid
R6G: rhodamine 6G
SE(R)RS: surface-enhanced (resonance) Raman spectroscopy
SEF: surface enhancement factor
TFF: tangential flow filtration.
