# Supplementary material for: Ecofriendly Filtration of Silver Nanoparticles for Ultrasensitive Surface-Enhanced (Resonance) Raman Spectroscopy-Based Detection
Source: J Phys Chem C Nanomater Interfaces. 2024 Sep 23;128(39):16563–75. doi: 10.1021/acs.jpcc.4c03837 (PMC11457218; doi:10.1021/acs.jpcc.4c03837)
Supplement: Supplementary file 1 — jp4c03837_si_001.pdf [file jp4c03837_si_001.pdf]

SUPPORTING INFORMATION

"Ecofriendly Filtration of Silver Nanoparticles for Ultrasensitive Surface-Enhanced (Resonance) Raman Spectroscopy-Based Detection"

*Kevin M. Dorney,[1] Nicholas S. Shropshire,[2] Daniel G. Adams,[2] Ashkan Zandi,[3] Joshua Baker,[1] Seth Brittle,[1] Sushil Kanel,[1,4] Nasrin Hooshmand,[2] and Ioana E. Pavel[2]**

[1]Department of Chemistry, Wright State University, 3640 Colonel Glenn Hwy., Dayton, OH 45435, United States

[2]Department of Physical and Environmental Sciences, Texas A&M University – Corpus Christi, Corpus Christi, TX 78412, United States

[3]School of Electrical and Computer Engineering, Georgia Institute of Technology, North Avenue, Atlanta, GA 30332, United States

[4]UES, 4401 Dayton Xenia Rd, Beavercreek, OH 45432, United States

Physicochemical Properties of AgNPs

Figure S1 shows two tangential flow filtration (TFF) systems: a commercial one and an in-house built one, which are utilized in our experiments for the size-selection, purification, and concentration of colloidal silver nanoparticles (AgNPs).

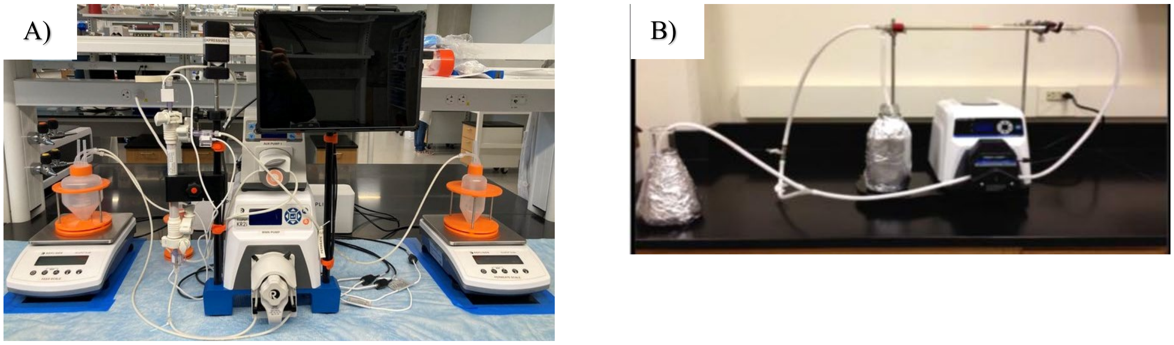

Figure S1. Photos of two illustrative tangential flow filtration (TFF) systems: **A)** a commercial KrosFlo KR2i TFF system (Repligen Inc.) and **B)** an in-house, simplified TFF system.

Figure S2 displays the ICP-OES external calibration curves that were utilized for the quantification of the total silver (Ag) and sodium (Na) content of each colloidal sample through interpolation. Matrix effects were found to be negligible in these colloidal AgNPs through the standard addition methods (relative percent differences between the two methods <10%) [1-4].

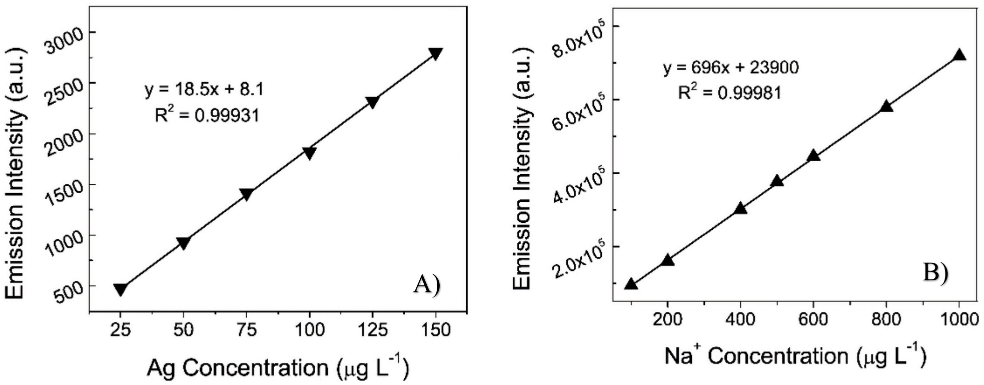

Figure S2. ICP-OES external calibration curves constructed for the total **A)** silver (Ag) and **B)** sodium (Na) quantifications of the colloidal samples of Creighton silver nanoparticles (AgNPs). The following six (6) Ag external standards and one blank were considered: 0, 25, 50, 75, 100, 125, and 150 μg L^{-1}. The following seven (7) Na external standards and one blank were utilized: 0, 100, 200, 400, 500, 600, 800, and 1,000 μg L^{-1}.

Table S1 summarizes the total Ag and Na content of each colloidal samples before and after the TFF manipulation of Creighton AgNPs. The total Ag content was measured after diluting all TFF aliquots to concentrations consistent with the lowest yield of the TFF-based separation processes (n = 3 independent experiments).

Tables S1. The total silver (Ag) and sodium (Na) content as determined by ICP-OES for each colloidal samples of AgNPs and controls (blanks, no metal) before and after TFF in n = 3 independent experiments.

Colloid Sample	Total Ag^+ (μg mL^{-1})[a]	Total Na^+ (μg mL^{-1})
Original Colloid (ORI)	10.1 ± 0.4	40.5 ± 9.8
50 nm Retentate (AgNP≥40)	106.8 ± 0.9	15.4 ± 1.8
50 nm Filtrate	8.1 ± 0.1	41.1 ± 7.4
1st 30 kDa Retentate	107.8 ± 3.4	28.1 ± 2.1
1st 30 kDa Filtrate	0.52 ± 0.01	36.9 ± 10.3
2nd 30 kDa Retentate (AgNP ≤40)	899.9 ± 6.1	18.6 ± 1.4
2nd 30 kDa Filtrate	0.57 ± 0.02	28.7 ± 6.1

[a]The total silver content was determined after diluting the TFF colloids to concentrations consistent with the lowest yield of the three separation processes.

Table S2 summarizes the chemical (silver content – Conc.), morphological (average diameter – Ave. Dia., maximum diameter – Max. Dia., and % distribution of diameters), and optical (localized surface plasmon resonance (LSPR) maximum - λ_{max}) properties of quasi-spherical, unfunctionalized AgNPs before (ORI) and after tangential flow filtration (AgNP≥40 and AgNP≤40).

Table S2. Morphological, optical, and chemical properties of the original colloid (ORI) and the two representative TFF-fractions (AgNP≥40 and AgNP≤40) utilized in the SE(R)RS experiments.

Sample	ORI	AgNP≥40	AgNP≤40
Conc. (µg mL^{-1})	10.1	106.8	899.9
Ave. Dia. (nm)	13.4	20.1	12.4
Max. Dia. (nm)	167.4	77	41.4
% Distribution	105.5	64	77
λ_{max} (nm)	393	403	387

SE(R)RS of R6G using colloidal AgNPs from ORI, Ag50R, and Ag30R2

Preparation of rhodamine 6G (R6G) and SE(R)RS samples

First, an aqueous stock solution of 10^{-3} M of R6G (Fisher Scientific, CAS#989-38-8) was prepared in HQ H_2O (> 18 MΩ cm). R6G aqueous solutions of 10^{-5}–10^{-14} M were then obtained through serial dilutions of the R6G stock solution, and the R6G concentration of each solution was confirmed spectrophotometrically via the absorption maximum of R6G at ~528 nm. Each SE(R)RS sample was prepared by adding 400 µL of the R6G solution, 2 mL of AgNPs, 1.4 mL of HQ H_2O, and ~200 µL of 1.0 M KBr in water [5]. Samples were allowed to incubate for 24 hours at room temperature to ensure the effective adsorption of R6G to AgNPs and the formation of SE(R)RS-active "hot-spots" before spectral analysis. SERS and SERRS samples were prepared separately to prevent any carry-over effects of photo-induced degradation of R6G between sample measurements [6].

Cleaning Procedures for Single-Molecule (SM) Detection Events

In aqueous solutions near a neutral pH, R6G acts as a cationic dye species due to a positively charged imine bond. The positive charge of R6G cations may result in an adsorption equilibrium with both the negatively charged AgNPs (-28±2 mV at pH of 6.8 [4]) and the partially negative dioxide groups on the cuvette walls. Therefore, extensive cleaning procedures were employed to remove any R6G physically adsorbed on to the walls of the cuvette. Substrate “blanks” (*i.e.*, SE(R)RS samples containing only AgNPs and KBr but no R6G) were analyzed in between each SERRS and SM-SERRS sample to ensure there was no cross-over R6G. If R6G was detected in the blank spectra, cuvettes were thoroughly rinsed with ethanol and heated at 500 °C for 24 hours. Following thermal degradation of R6G, cuvettes were placed in a 3:1 v/v $HCl:HNO_3$ solution for 1 hour, followed by thorough washing with HQ H_2O and storage in ethanol. Blank SE(R)RS samples were again analyzed in the cleaned cuvette to confirm the complete removal of R6G.

Raman and SE(R)RS Data

Figure S3 presents the Raman control spectra of the three colloidal samples utilized in the SE(R)RS experiments: the original colloid (ORI) and the two tangential flow filtration (TFF) fractions, AgNP≥40 and AgNP≤40. Herein, only KBr (no R6G probe) was added to promote the formation of SERS hot spots.

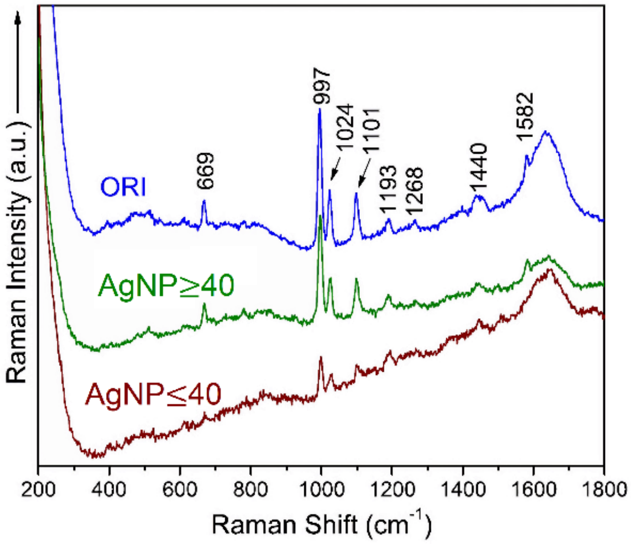

Figure S3. Raman control spectra of the original colloid (ORI) and the two TFF fractions, AgNP≥40 and AgNP≤40, in the presence of only KBr. The intensity scale is identical in each spectrum, with the only modification being an intensity axis off-set for clarity.

The SE(R)RS spectrum of R6G adsorbed on each colloidal sample of AgNPs was recorded in a concentration dependent manner to accurately determine the effects of AgNP size, total silver concentration, and excitation wavelength on the SE(R)RS signal enhancements. Samples with R6G concentrations ranging from 10^{-6} M to 10^{-9} M were utilized in the SERS measurements, while SERRS and SM-SERRS measurements were performed using R6G concentrations of 10^{-8} M to 10^{-15} M. The R6G:AgNPs ratios were well below 3:1 for both excitation lines at 532.1 nm and 632.8 nm. This helped avoid any deviations in the calculated surface enhancement factors (SEFs) associated with the over-saturation of R6G [7]. The SERS, SERRS, and SM-SERRS spectra of R6G adsorbed on ORI, AgNP≥40, and AgNP≤40, are presented in Figures S4, S5, and S6, respectively. The unaltered spectra were shifted on the Y axis for comparison purposes in these figures (Figures S4-S6).

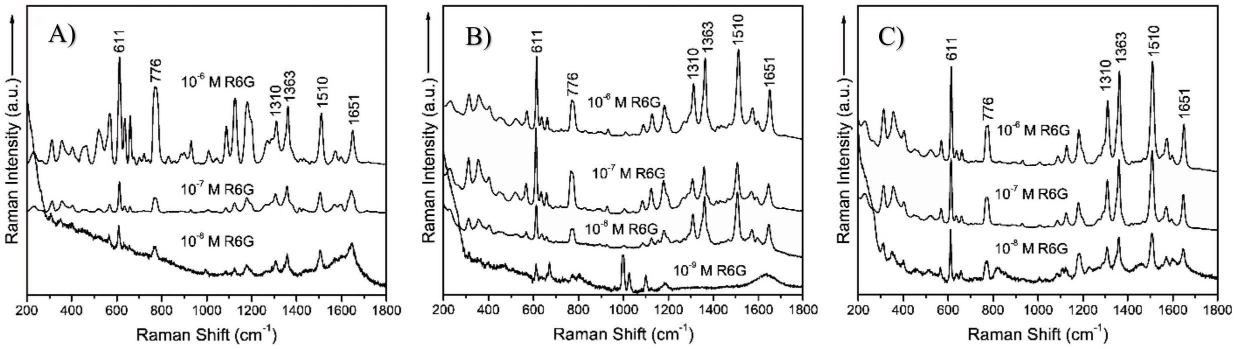

Figure S4. SERS spectra of R6G collected in the 10^{-6}-10^{-9} M concentration range using a 632.8 nm excitation laser and the following colloidal samples of AgNPs: **A)** ORI, **B)** AgNP≥40, and **C)** AgNP≤40.

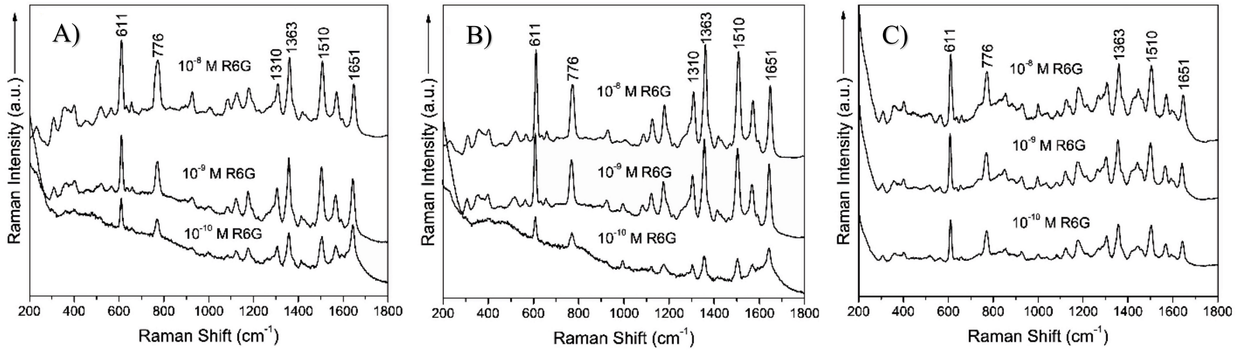

Figure S5. SERRS spectra of R6G collected in the 10^{-8}–10^{-10} M concentration range using a 532.1 nm excitation laser and the following colloidal samples of AgNPs: **A)** ORI, **B)** AgNP≥40, and **C)** AgNP≤40.

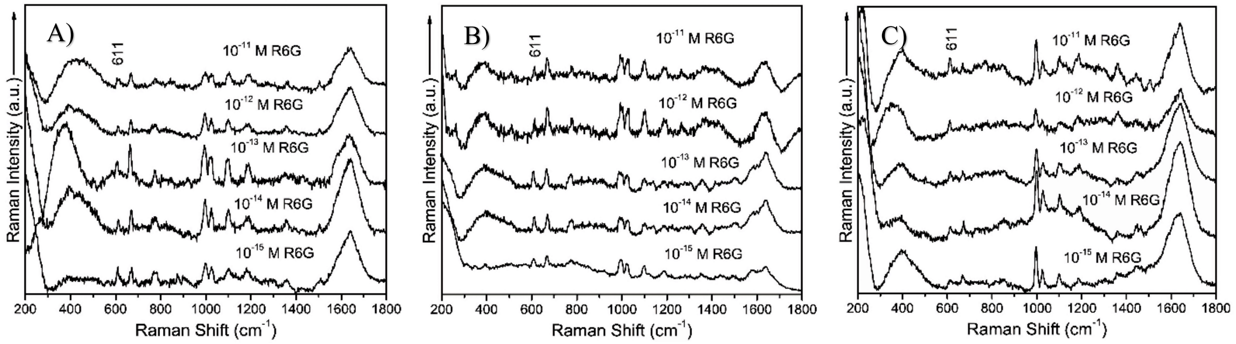

Figure S6. SM-SERRS spectra of R6G collected in the single molecule regime (10^{-11}–10^{-15} M) using a 532.1 nm excitation laser and the following colloidal samples of AgNPs: **A)** ORI, **B)** AgNP≥40, and **C)** AgNP≤40.

Percentage of R6G Adsorbed onto AgNPs in SE(R)RS Samples

The fluorescence emission of R6G is readily quenched when R6G molecules are adsorbed to or located within the immediate vicinity of AgNPs. Thus, differential fluorescence spectrophotometry can estimate the amount of R6G bound to AgNPs in suspension. The fluorescence emission of aqueous R6G was recorded using a Cary Eclipse Fluorescence Spectrophotometer (Agilent Technologies, Inc.) at 530 nm excitation and 545 nm emission. Aqueous stock solutions of 10^{-6}–10^{-8} M of R6G were prepared and analyzed for overall emission intensity. SE(R)RS samples of the same concentrations were prepared and allowed to incubate in the absence of light, for 24 hours, prior to the fluorescence analysis. Following incubation, samples were centrifuged at 7.8 x 10^3 g for 1 hour, and the resulting supernatant was extracted and analyzed for the fluorescence emission of R6G (Figure S7). In addition, SE(R)RS samples were prepared by using AgNP≥40 and AgNP≤40 colloids of a nanosilver concentration equal to that of ORI to determine concentration dependent absorption behavior.

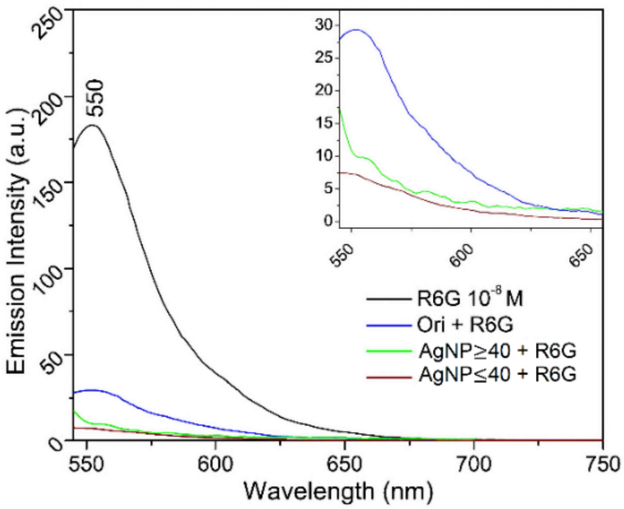

Figure S7. Representative fluorescence emission spectra of a bulk solution of 10^{-8} M of R6G and the SE(R)RS samples at 10^{-8} M of R6G (denoted ORI+R6G, AgNP≥40+R6G, and AgNP≤40+R6G). Inset shows the expanded emission region of the supernatant of the SE(R)RS samples collected through centrifugation.

The difference in fluorescence emission intensities between the bulk solution and the SE(R)RS supernatant is directly related to the amount of R6G adsorbed onto AgNPs. The supernatant contains “free” R6G molecules (R6G-solution) and is separated from the R6G molecules complexed to AgNPs (R6G-AgNP in the centrifuge pellet) by centrifugation. The amount of R6G adsorbed onto single or aggregates of AgNPs (dimer, trimers, etc.) can be quantified (equation 1) through the difference in fluorescence emission of R6G before (“free” R6G) and after (adsorbed R6G-AgNP) incubation with the colloidal samples of AgNPs as follows:

$$C_{R6G-AgNP} = C_{R6G-solution}\left[1-\left(\frac{I_{R6G,SE(R)RS\ supernatant}}{I_{R6G,solution}}\right)\right] \quad (1),$$

where C and I denote the concentrations and the fluorescence intensities of R6G in the bulk solution and SE(R)RS sample, respectively.

Table S3. Fluorescence emission data and calculated adsorption ratios of R6G in SE(R)RS.

Substrate	$C_{R6G\text{-}Solution}$ (M)	$I_{R6G,solution}$ (a.u.)[a]	$I_{R6G,\ SE(R)RS}$ (a.u.)[a]	% Absorbed R6G[b]
ORI	1 x 10^{-6}	663	162	77.8 ± 2.2
	1 x 10^{-7}	340	62	82.4 ± 0.3
	1 x 10^{-8}	182	29	84.1 ± 0.1
AgNP≥40	1 x 10^{-6}	663	60	91.2 ± 1.1
	1 x 10^{-7}	340	29	92.4 ± 1.4
	1 x 10^{-8}	182	6	93.7 ± 1.2
AgNP≤40	1 x 10^{-6}	663	127	92.1 ± 0.4
	1 x 10^{-7}	340	43	92.9 ± 5.0
	1 x 10^{-8}	182	10	95.9 ± 1.4
AgNP≥40[c]	1 x 10^{-6}	442	156	62.4 ± 2.0
	1 x 10^{-7}	260	97	64.4 ± 1.7
	1 x 10^{-8}	70	16	74.0 ± 3.2
AgNP≤40[c]	1 x 10^{-6}	442	135	67.1 ± 2.4
	1 x 10^{-7}	260	51	77.0 ± 1.7
	1 x 10^{-8}	70	9	83.6 ± 3.6

[a]Representative fluorescence emission data for one group of TFF-fractionated colloids.

[b]Average R6G absorption ratios for three different TFF separation experiments.

[c]Samples were diluted to match the metal concentration of ORI at 10.1 μg mL^{-1}.

As one would expect, the largest percent of adsorbed R6G molecules was observed for the AgNP≤40 samples at all concentrations (Table S3). This is due to the smaller size, larger silver concentration, and higher total surface area available for analyte adsorption in AgNP≤40 than in AgNP≥40 (Table S2). The fluorescence of R6G was undetectable in stock solutions at 10^{-9} M or lower. Therefore, the percent of R6G molecules adsorbed at 10^{-8} M in the SE(R)RS samples was utilized for the SEF calculations in the 10^{-9}–10^{-15} M concentration range.

Number of Bulk AgNPs and R6G:AgNP Complexes within the Laser Focal Volumes

The results of the TEM and ICP-OES analyses provided corroborating data that allowed for the determination of the key physicochemical properties of AgNPs. Most notably, the concentration of nanosilver, average diameter of AgNPs, and volume of ORI, AgNP≥40 and AgNP≤40, facilitated the extrapolation of the number of AgNPs present in each SE(R)RS sample. If the atomic radius of silver is known [8] and the AgNPs are predominately quasi-spherical (Creighton synthesis), the number of AgNPs within the SE(R)RS sample can be stoichiometrically calculated. An example calculation is provided below for the AgNP≥40 sample (equations 2-5).

$$V_{single\ AgNP} = \frac{4}{3} \cdot \pi \cdot (1.05 \times 10^{-8} m)^3 = 4.25 \times 10^{-24} m^3 \qquad (2),$$

$$V_{Atomic\ silver} = \frac{4}{3} \cdot \pi \cdot (1.6 \times 10^{-10} m)^3 = 1.72 \times 10^{-29} m^3 \qquad (3),$$

$$N_{Ag\ atoms\ per\ AgNP} = \left(\frac{4.25\times10^{-24} m^3}{1.72\times10^{-29} m^3}\right) = 247{,}821\ \frac{Ag\ atoms}{AgNP} \qquad \text{(4), and}$$

$$N_{AgNPs,SE(R)RS} = \left(\frac{106.8\ \text{mg of Ag}}{1\ L}\right) \times (0.06\ L) \times \left(\frac{1\ g}{1{,}000\ mg}\right) \times \left(\frac{1\ mol\ of\ Ag}{107.8682\ g\ of\ Ag}\right) \times$$

$$\left(\frac{6.022\times10^{23} Ag\ atoms}{1\ mol}\right) \times \left(\frac{1\ AgNP}{247{,}821\ Ag\ atoms}\right) \times \left(\frac{0.002\ L\ for\ SE(R)RS}{0.06\ L\ \ for\ solution}\right) = 4.81 \times 10^{12} AgNPs \qquad (5),$$

where *V* and *N* are abbreviations for volume and number.

Similar calculations were performed for ORI and AgNP≤40. The results are summarized in Table S4 along with the number of R6G molecules and the molar concentration ratio of R6G:AgNP for the most concentrated sample of AgNPs utilized in this study.

Table S4. Number of AgNPs and R6G molecules adsorbed onto AgNPs in the SE(R)RS colloidal volume (2 mL) at the highest concentration of R6G at 10^{-6} M.

SE(R)RS Substrate	Number of AgNPs	Number of R6G Molecules	[R6G]:[AgNP]
ORI	1.6×10^{12}	8.0×10^{15}	1.2×10^{-2}
AgNP≥40	4.8×10^{12}	5.5×10^{17}	1.0×10^{-3}
AgNP≤40	1.7×10^{14}	4.9×10^{17}	1.2×10^{-4}

A further extension of the treatment presented in the previous equations can be utilized to determine the total surface area available in the SE(R)RS samples (2 mL) for analyte adsorption. The surface area (*S.A.*) of a single AgNP can be estimated using known geometric arguments and the observed TEM diameters. Herein, it is assumed that all AgNPs are spherical or nearly spherical with aspect ratios of 1.0–1.2. The *S.A.* per AgNP and the total *S.A.* within each SE(R)RS sample are presented in Table S5.

Table S5. Surface areas (*S.A.*) for a single AgNP and all AgNPs in the SE(R)RS samples.

SE(R)RS Substrate	S.A. Single AgNP (m^2)	S.A. SE(R)RS Sample (m^2)
ORI	5.64×10^{-16}	8.92×10^{-4}
AgNP≥40	1.27×10^{-15}	6.1×10^{-3}
AgNP≤40	4.83×10^{-16}	2.50
AgNP≥40[a]	1.27×10^{-15}	5.94×10^{-4}
AgNP≤40[a]	4.83×10^{-16}	9.64×10^{-4}

[a]Diluted to match the concentration of ORI at 10.1 µg mL^{-1}.

Optical diffraction theory allows for an estimation of the focal volume (*F.V.* – equation 6) for a given laser source passing through a focus lens [9,10]:

$$F.V. = \pi \cdot \left(\frac{1.22\lambda}{2NA}\right)^2 \cdot \left(\frac{4\lambda}{NA^2}\right) \quad (6),$$

where λ is the excitation wavelength in meters (m) and *NA* is the numerical aperture of the objective lens (0.5 for the 50X Olympus objective utilized in the SE(R)RS measurements). Using the two excitation wavelengths in this study (632.8 nm and 532.1 nm), the focal volumes were estimated to be 1.9×10^{-14} L and 1.13×10^{-14} L, respectively. Lastly, the total number of AgNPs that are present within the laser focal volume for each SE(R)RS sample can be statistically estimated via the following formula:

$$AgNPs_{F.V.} = \left(\frac{N_{AgNPs,SE(R)RS}}{V_{SE(R)RS\,(L)}}\right) \cdot V_{F.V.}(L) \qquad (7),$$

where *N* is the number of AgNPs within the SE(R)RS sample, *V* refers to the volume of the SE(R)RS sample and *FV* denotes the focal volume in liters (L). The number of AgNPs and R6G

molecules located within the *F.V.* for each excitation wavelength are summarized in Table S6. These results indicate a significantly larger number of AgNPs when compared to the number R6G molecules in the SM-SERRS measurements.

Table S6. Estimated number of AgNPs and R6G molecules within the laser focal volume (*F.V.*) for SERS and SERRS measurements.

SE(R)RS Substrate	SERS (λ_{ex} = 632.8 nm)	SERRS (λ_{ex} = 532.1 nm)
ORI	~ 15	~ 9
AgNP≥40	~ 46	~ 27
AgNP≤40	~ 1637	~ 973
R6G	~ 1[a]	~ 1[b]

Calculated for samples containing [a]1.0 x 10^{-10} M and [b]5.0 x 10^{-10} M of R6G molecules.

Figure S8 displays the SERS intensities that were recorded under the pre-resonance excitation of R6G (632.8 nm), after the dilution of the two TFF fractions down to the metal concentration of (ORI of 10.1 µg mL^{-1}).

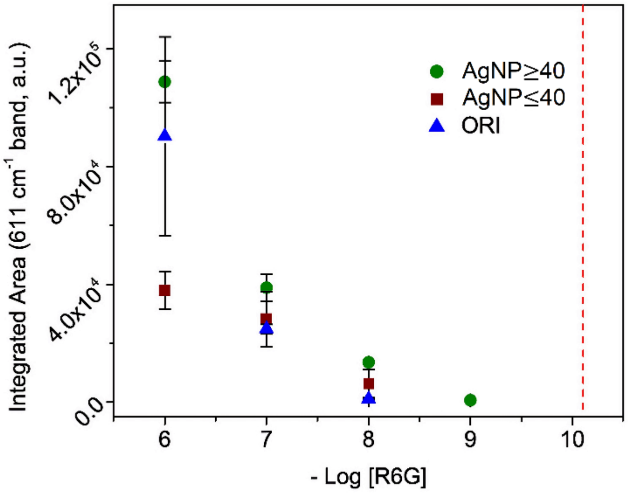

Figure S8. Plot of the integrated area of the 611 cm^{-1} marker band of R6G versus the negative log of R6G concentration obtained after the incubation with ORI and the two diluted TFF fractions. The metal concentration was the same in all colloidal samples (ORI, AgNP≥40, and AgNP≤40): 10.1 µg mL^{-1}.

Figure S9 displays the TEM images of the AgNP≥40 fraction of an average diameter of 20.1 nm before (106.8 μg mL^{-1}) and after dilution (10.1 μg mL^{-1}) with water. This facilitated the comparison of SER(R)S-based detection efficiency of the diluted AgNP≥40 fraction to that of ORI at the same concentration of ORI (10.1 μg mL^{-1}). Below the SM threshold (10^{-11}-10^{-15} M of R6G), the SM-SERRS detection of R6G was favored by the larger AgNPs in the AgNP≥40 fraction with or without the dilution of AgNPs. As expected, only a small percentage of the observed AgNPs in the diluted fraction of AgNP≥40 were represented by dimers or other small aggregates. This is probably due to the very small number of R6G cationic molecules that are available for creating SERS "hot spots" through electrostatic bridges with the negatively charged AgNPs (e.g., $AgNP^-$-$R6G^+$-$AgNP^-$ dimers). In fact, there is statistically about one R6G molecule present within the focal volume at a R6G concentration of 1.0-5.0 x 10^{-10} M. Overall, AgNP≥40 gave rise to the largest SEF values (2.5 x 10^{10}) and its concentration was found to play a minute role under single molecule (SM) resonance excitation at 532.1 nm.

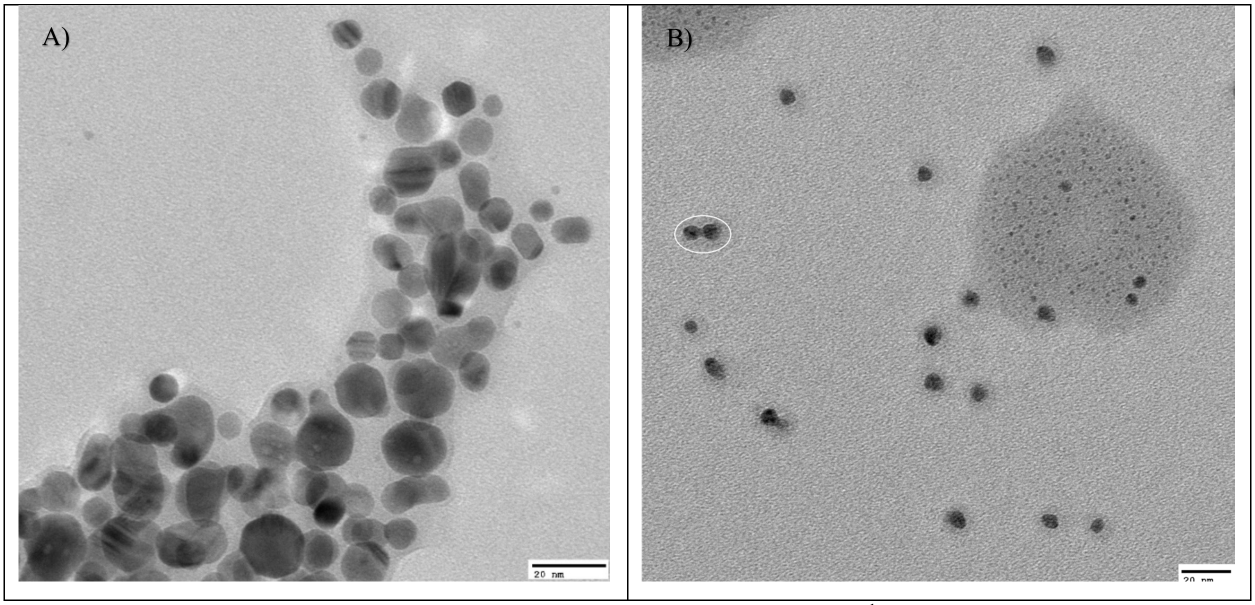

Figure S9. TEM micrographs of the **A)** concentrated (106.8 μg mL^{-1}) and **B)** diluted (10.1 μg mL^{-1}) fractions of AgNP≥40 for SER(R)S with R6G test probe. A) The silver nanoparticles (AgNPs) have an average diameter of 20.1 nm and appear black on a lighter grey background. B) At R6G concentrations (10^{-11}-10^{-15} M of R6G) close to or below the single-molecule (SM) regime, only a small percentage of the observed AgNPs in the diluted fraction of AgNP≥40 were represented by dimers or other small aggregates (dimer example enclosed by a white circle). Scale bar is 20 nm.

References

[1] Trefry, J. C.; Monahan, J. L.; Meyerhoefer, A. J.; Markopolous, M. M.; Arnold, Z. S.; Wooley, D. P.; Pavel, I. E. Size Selection and Concentration of Silver Nanoparticles by Tangential Flow Ultrafiltration for SERS-Based Biosensors. *J. Am. Chem. Soc.* **2010**, *132* (32), 10970-10972. DOI: 10.1021/ja103809c

[2] Dorney, K. M.; Baker, J. D.; Edwards, M. L.; Kanel, S. R.; O'Malley, M.; Pavel (Sizemore), I. E. Tangential Flow Filtration of Colloidal Silver Nanoparticles: A "Green" Laboratory Experiment for Chemistry and Engineering Students. *J. Chem. Ed.* **2014,** *91*, 1044-1049. DOI: 10.1021/ed40068u

[3] Wells, K. L.; Alla, P. K.; Kaiser, K. G.; Murgulet, I. T.; Adragna, N. C.; Pavel, I. E. SERS of Human Red Blood Cells in Non-Resonant Conditions: Benefits, Limitations, and Complementary Tools (CytoViva and GFAAS). *Chemosensors* **2023**, *11:353*, 1-17. DOI: 10.3390/chemosensors11070353

[4] Paluri, S. L. A.; Ryan, J. R.; Lam, N. H.; Nepal, D., Pavel (Sizemore), I. E. Analytical-Based Methodologies for Examining the *In Vitro* Absorption, Distribution, Metabolism, and Elimination (ADME) of Silver Nanoparticles. *Small* **2017**, *13:1603093*, 1-15. DOI: 10.1002/smll.201603093

[5] Hildebrant, P.; Stockburger, M. Surface-enhanced resonance Raman spectroscopy of Rhodamine 6G adsorbed on colloidal silver. *J. Phys. Chem.* **1984**, *88* (24), 5935-5944. DOI: 10.1021/j50668a038

[6] Emory, S. R.; Ambrose, W. P.; Goodwin, P. M.; Keller, R. A. Observing single-molecule chemical reactions on metal nanoparticles. *Proc. SPIE* **2001**, *4258*, 63-72. DOI: 10.1117/12.430762

[7] Kneipp, K.; Wang, Y.; Dasari, R. R.; Feld, M. S. Approach to Single Molecule Detection Using Surface-Enhanced Resonance Raman Scattering (SERRS): A Study Using Rhodamine 6G on Colloidal Silver. *Appl. Spectrosc.* **1995**, *49* (6), 780-784. DOI: 10.1366/0003702953964480

[8] Slater, J. C. Atomic Radii in Crystals. *J. Chem. Phys.* **1964**, *41(10)*, 3199-3204. DOI: 10.1063/1.1725697

[9] Everall, N. J. Modeling and Measuring the Effect of Refraction on the Depth Resolution of Confocal Raman Microscopy. *Appl. Spectrosc.* **2000**, *54*, 773-782. DOI: 10.1366/0003702001950382
[10] Everall, N. J. Confocal Raman Microscopy: Why the Depth Resolution and Spatial Accuracy Can Be Much Worse Than You Think. *Appl. Spectrosc.* **2000**, *54*, 1515-1520. DOI: 10.1366/0003702001948439
